# Prospective study of oil spill cleanup-related exposure to volatile organic compounds and glycemic dysregulation

**DOI:** 10.1186/s12940-025-01211-5

**Published:** 2025-09-26

**Authors:** Hanna V. Jardel, Alex P. Keil, Chantel L. Martin, David B. Richardson, Mark R. Stenzel, Patricia A. Stewart, Kate E. Christenbury, Dale P. Sandler, Lawrence S. Engel

**Affiliations:** 1 Department of Epidemiology, Gillings School of Global Public Health, University of North Carolina at Chapel Hill, Chapel Hill, NC USA; 2 Epidemiology Branch, National Institute of Environmental Health Sciences, Research Triangle Park, NC USA; 3 Occupational and Environmental Epidemiology Branch, Division of Cancer Epidemiology and Genetics, National Cancer Institute, Bethesda, MD USA; 4 Carolina Population Center, University of North Carolina at Chapel Hill, Chapel Hill, NC USA; 5 Department of Environmental and Occupational Health, Joe C. Wen School of Population & Public Health, University of California, Irvine, CA USA; 6 Exposure Assessment Applications, LLC, Arlington, VA USA; 7 Stewart Exposure Assessments, LLC, Arlington, VA USA; 8 DLH, LLC, Bethesda, MD USA

**Keywords:** HbA1c, Diabetes, BTEX, Mixture, Oil spill cleanup, Quantile g-computation

## Abstract

**Background:**

Exposures to volatile organic compounds could influence glycemic regulation. This study examines hemoglobin A1c (HbA1c) in a cohort of oil spill cleanup workers up to 6 years post-exposure in relation to benzene, toluene, ethylbenzene, and xylenes (BTEX) exposures, individually and as a mixture, as well as a separate estimation of the aggregate sum of BTEX (total BTEX).

**Methods:**

Data for this analysis are from the Gulf Long-term Follow-up (GuLF) Study– a prospective cohort of workers involved in the 2010 *Deepwater Horizon* oil spill cleanup. HbA1c and medication information were obtained at Home Visit and Clinical Exam phases 1–3 years and up to 6 years post-exposure, respectively. Cumulative inhalation exposure to the individual BTEX chemicals and to total BTEX were estimated using a job-exposure matrix linking air measurements to detailed individual worker cleanup work histories. We used Tobit regression models to examine associations between exposure to the chemicals and latent, untreated HbA1c, accounting for medication-reduced HbA1c. We used quantile g-computation to examine exposure to the mixture of BTEX chemicals and HbA1c.

**Results:**

In results examining Home Visit HbA1c we observed no discernable patterns but found suggestive evidence of an association with total BTEX. In results for Clinical Exam HbA1c, we did not observe monotonic patterns, but rather an inverted-U pattern with elevations in Q2 or Q3 or no clear pattern. Similarly, in results for final HbA1c adjusting for initial HbA1c, total BTEX difference estimates showed an inverted-U pattern in point estimates across Q2 (0.24 95%CI (0.14, 0.34)), Q3 (0.13 95%CI (0.03, 0.24)), and Q4 (0.00 95% CI (-0.11, 0.10)), compared to Q1.

**Conclusion:**

Exposures to the moderate levels of the BTEX chemicals observed in this study population, individually and as an aggregate, may be associated with elevated HbA1c up to 6 years after exposure, with an inverted-U pattern.

**Supplementary Information:**

The online version contains supplementary material available at 10.1186/s12940-025-01211-5.

## Introduction

Despite the considerable number of large oil spills, there have been few studies on the long-term health effects of specific oil spill exposures: between 1970 and 2019 there were 467 oil tanker spills that each spilled over 700 tons of oil [1, 2]. Individuals who clean up spilled crude oil may experience health effects related to their exposure to highly volatile organic compounds and other, less volatile, hydrocarbons (e.g., paraffins). Volatile organic compounds of particular concern include benzene, toluene, ethylbenzene, and xylenes (*m-*,* p-*,* o*-)(BTEX). Exposure to the BTEX pollutants may contribute to the risk of developing subclinical and, eventually, clinically relevant physiological changes indicative of chronic disease such as diabetes mellitus [3–9].

Glycemic dysregulation is clinically known as diabetes mellitus (DM) [10]. Hemoglobin A1c (HbA1c) is a biomarker of glycemic regulation [11–13]. Clinically, a HbA1c value greater than or equal to 6.5% (percent of hemoglobin with a glucose attached) is considered indicative of diabetes [10]. Non-white individuals and those of lower socioeconomic status are more likely to develop diabetes [14–17], potentially through increased chronic stress due to living in more socioeconomically deprived areas [18], or in the US, being identified as a race other than White [19, 20]. Known risk factors include obesity, low levels of activity, and family history of diabetes [21]. Environmental exposures are known contributors to DM, yet little is known about the relationship to BTEX exposure specifically.

Previous work in the GuLF Study suggested associations between increasing total hydrocarbon exposure (which includes about 10% by weight of BTEX [22]) and increased risk of incident DM [6]. In the general population, increased levels of blood benzene, xylenes, and ethylbenzene have been associated individually with increased levels of HbA1c [4]. Benzene is the best studied of the BTEX chemicals in relation to diabetes risk; urinary levels of benzene metabolites have been associated with increased insulin resistance [3, 5] and increased risk of diabetes [7] in the general population. Multiple years of exposure to both ethylbenzene and styrene together has been associated with increased blood glucose levels [9]. Paint factory workers exposed to toluene, xylenes, and styrene were shown to have higher fasting glucose and insulin resistance [8]. There is some evidence to suggest that oxidative stress likely plays a role in these results.

The key hormone in glycemic regulation is insulin. Insulin is secreted by pancreatic β-cells and signals to other cells in the body to take in glucose from the blood. Oxidative stress has been associated with both the reduced production of insulin as well as reduced somatic cell sensitivity to insulin and with DM overall; it has been suggested that somatic cell insulin resistance perpetuates itself through hyperglycemia and increased β-cell oxidative stress, which lead to further somatic cell insulin resistance [23–26].

Exposure to BTEX may induce oxidative stress, as supported by work involving humans, human in vitro assays, and rodent models, and has been separately linked to increased oxidative stress markers, oxidative DNA damage, and altered gene expression [27–39]. Oxidative stress is linked with subclinical processes that are related to feedback loops in DM and β-cell function [23, 24, 40–42] as well as overall metabolic function [25, 43–49]. This may be through disruption of normal β -cell internal signaling (which uses oxidative molecules to down-regulate insulin production [41] or through direct damage to the cell DNA (which β-cells are known to be inefficient at repairing) [40]. As reviewed by Pi et al. (2010) [42], oxidative stress compensation mechanisms also reduce β-cell capacity to secrete insulin; chronic excess of ROS could cause prolonged β-cell dysfunction.

This analysis is the first to examine the BTEX chemicals in relation to HbA1c among oil spill cleanup workers, a group of people who had the potential to experience higher exposures than the general population. Additionally, it is the first to examine the BTEX chemicals as a mixture in relation to HbA1c. It relates these exposures to HbA1c at two time points up to 6 years apart after exposure. Additionally, it addresses left censoring of HbA1c due to HbA1c-lowering medication use using a censored linear regression.

## Methods

### Gulf study

The GuLF Study is a prospective cohort consisting of individuals over 21 years old at baseline (2011–2013) who worked on the 2010 *Deepwater Horizon* (*DWH*) oil spill cleanup and response or who completed cleanup safety training but did not participate in the cleanup [50]. The GuLF Study cohort is the largest prospective study (*n* = 32, 608 at enrollment) to date of oil spill cleanup workers with detailed exposure and outcome data [50]. A total of 11,193 English- or Spanish-speaking individuals—8,968 of whom were cleanup workers—who resided in states bordering the Gulf of Mexico completed both a telephone enrollment interview and a Home Visit that included collection of biologic samples 1–3 years following the spill (2011–2013). Of everyone who completed the Home Visit, 3,401 eligible participants residing within approximately 60 miles from a study clinic in Mobile, AL or New Orleans, LA completed a Clinical Exam about 3 years later (2014–2016).

**Fig. 1:**
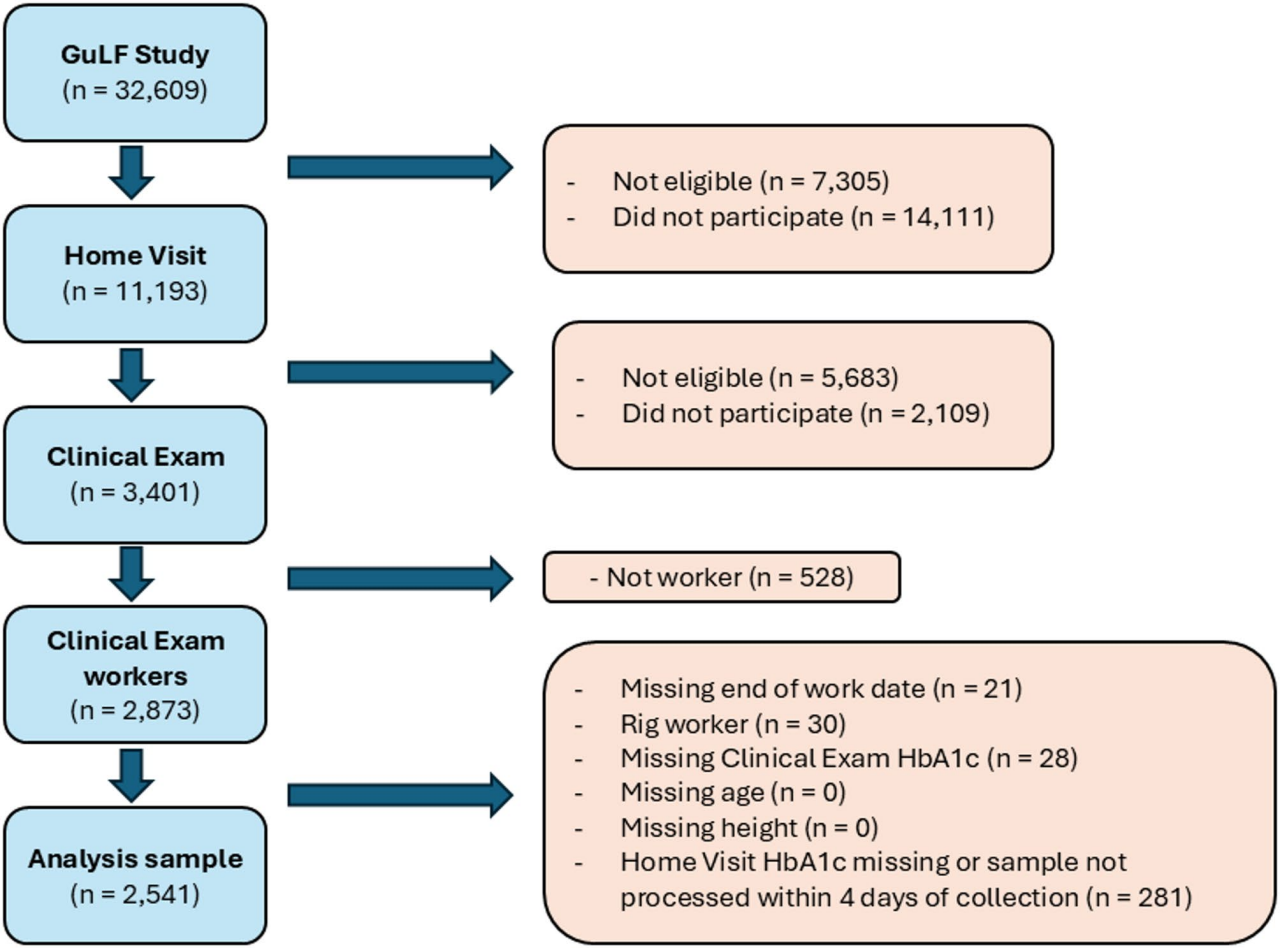
Flowchart of analysis sample inclusion

Of the whole cohort 2,873 individuals who worked on the oil spill clean-up for at least 1 day (workers) and participated in both the Home Visit and Clinical Exam were eligible for this analysis (see Fig. 1). We excluded those *(i)* with did not have more than 1 aliquot of Home Visit specimen processed within 4 days after collection (*n* = 281), *(ii)* without a Clinical Exam reading (*n* = 28), *(iii)* without complete information on key covariates (age, sex, height, date of end of work on the spill) (*n* = 21). We excluded those with biospecimens processed more than 4 days after collection due to concerns about sample degradation and we retained the last aliquot of a given biospecimen for future analyses. We also excluded 30 participants who worked on one of the four rigs but otherwise met inclusion criteria because these workers, professionals trained for these specific tasks, were likely provided more effective personal protective equipment and safety training, which may have resulted in over-estimation of their received exposure. The Gulf Study was approved by the institutional review board of the National Institutes of Health. Verbal consent was collected for all telephone interview phases and written consent collected for all in-person phases.

### Exposure assessment

The process of estimating exposures is detailed elsewhere [51–55]. Briefly, the enrollment interview collected data on jobs/tasks/activities (hereafter called jobs) that participants reported working during the oil spill response and cleanup (OSRC), the spatiotemporal parameters of those jobs, and the context of those job (e.g., on land, on a boat, etc.) [55]. GuLF Study industrial hygienists used both personal exposure and area measurement data collected by a separate party for occupational exposure limit monitoring during the OSRC effort. First, approximately 28,000 full-shift passive dosimeter samples collected from the worker population (independent of the GuLF Study) yielded over 143,000 measurements of THC and BTEX. As many measurements were below the measurement methods’ limits of detection, we used correlations of each of the BTEX-H measurements with the associated (and higher percentage of detection) THC measurements and Bayesian statistics to estimate arithmetic averages (and other statistics) for each job measured [54]. Second, multigas meters mounted on 38 water vessels primarily within 5 miles of the wellhead yielded over 26 million short-term (i.e. seconds) readings. As many of these vessels were missing measurements in important time periods, we processed the area measurements to develop 495 full-shift “time-weighted” vessel-day THC averages that were considered comparable to the 894 THC personal measurements on those same vessels [52, 53]. Using the same correlational method as used for the personal measurements, the THC estimates were converted into BTEX-H estimates and using Bayesian statistics were included with those measurements to derive arithmetic means. Interview information (e.g., the tasks each person completed as part of their jobs, the job titles they had, when they worked each job, etc.) and measurements were used together to create a job-exposure matrix (JEM) that included over 3000 exposure groups, each group representing 1 or more jobs, with the members of each group expected to have a similar exposure distribution [55].

After reduction of air measurement censoring by recalculating exposure data to reflect analytical technique limits [55], industrial hygienists used Bayesian methods to estimate individual BTEX exposures in parts per billion (ppb) for each JEM exposure group [56]. From these estimates, total BTEX was calculated as the sum of the individual BTEX estimates (in ppb).

The industrial hygienists then assigned the JEM exposure group estimates to workers by linking them to individuals’ self-reported task information over the duration of their work on OSRC. Because we did not have information on how much time a worker spent on each job per day, if a worker indicated doing jobs that placed them in more than one exposure group during a given day, the highest exposure across all jobs for that day was assigned. These highest exposures were then summed over all days the individual worked on the spill to estimate the cumulative of the daily maximum. The majority (78%) of workers performed tasks associated with more than one exposure group (median 5 exposure groups, 25th percentile 2 exposure groups, 75th percentile 8 exposure groups). This analysis uses estimates of the cumulative of the daily maximum exposures that a worker experienced while working the 2010 D*WH* Oil Spill.

### Outcome assessment

The present study examined HbA1c at two time points: the Home Visit (*initial* HbA1c) and the Clinical Exam (*final* HbA1c) (see Supplementary Fig. 1). Average time between the two visits for the analysis sample was 37 months (standard deviation 7.7 months).

HbA1c at the Home Visit was ascertained at a National Glycohemoglobin Standardization Program reference lab (University of Minnesota) using packed red blood cells stored at -80 °C until analysis (in 2023). HbA1c has been measured in packed red blood cells previously [57, 58] and dilution series analysis shows that HbA1c levels are not affected by blood centrifugation and re-suspension [59]. HbA1c levels at the Clinical Exam were ascertained using a point-of-care device (DCA Vantage, Siemens Medical Solutions, Malvern, PA). Agreement between the two measurement techniques was excellent (intraclass correlation coefficient: 0.988 (95% CI 0.968, 0.995)) when HbA1c from 100 stored Clinical Exam samples were reassessed by the University of Minnesota laboratory. Other information included in the present analyses was participant use of prescription medications. Participants were requested to show study personnel all medications that they were currently taking at the time of the Home Visit and of the Clinical Exam and this information was recorded.

### Statistical methods

Exposures to each of the BTEX chemicals and total BTEX were represented as disjoint quartiles experienced by the overall analysis sample (Table 1). We did this to reduce the potential influence of exposure estimation error. We used both traditional single-pollutant Tobit regression and a novel Tobit form of quantile g-computation (QGcomp) [60] to estimate the mean difference and the corresponding 95% confidence intervals (95% CI) in percent latent, untreated HbA1c (% HbA1c) compared to Q1 pollutant % HbA1c. We used Tobit regression because it allowed us to address impacts of taking diabetes medication on HbA1c levels (see below) with less stringent modeling assumptions than would imputation-based methods (e.g., g-computation). QGcomp is a method designed to estimate effects of multiple exposures and we use it here to estimate the change in the mean HbA1c level for a simultaneous one-quartile increase of each mixture component (BTEX). These methods allowed us to interpret results as associations with latent, untreated HbA1c. We estimated associations between chemical exposures and initial HbA1c using both methods as follows (Eq. 1, for now, ignoring diabetes treatment):

1 $$\begin{array}{l}{HbA1c}_{initial}={\beta\:}_{0}+{\beta\:}_{1}\left(exposure\right)\\+{\beta\:}_{2}\left(duration1\right)+{\beta\:}_{z}\left(Z\right)+\epsilon\end{array}$$

Where *duration1* is the time between the end of exposure (end of cleanup work) and collection of Home Visit blood samples (initial HbA1c). We assessed associations between exposures and final HbA1c using both traditional Tobit regression and QGcomp with two separate models, one *not* adjusting for initial HbA1c (Eq. 2) and one adjusting for initial HbA1c (Eq. 3). We did this to ensure that regression to the mean did not induce patterns in equations controlling for initial HbA1c [61]. The equations are as follows:

2 $$\begin{array}{l}{HbA1c}_{final}={\beta\:}_{0}+{\beta\:}_{1}\left(exposure\right)+{\beta\:}_{2}\left(duration1\right)\\+\:{{\beta\:}_{3}\left(duration2\right)+\beta\:}_{z}\left(Z\right)+\epsilon\end{array}$$

3 $$\begin{array}{l}{HbA1c}_{final}={\beta\:}_{0}+{\beta\:}_{1}\left(exposure\right)+{\beta\:}_{2}\left({HbA1c}_{initial}\right)\\+{\beta\:}_{3}\left(duration1\right)+s\:{\beta\:}_{4}\left({HbA1c}_{initial}*\:duration1\right)\\+{{\beta\:}_{5}\left(duration2\right)+\beta\:}_{z}\left(Z\right)+\epsilon\end{array}$$

Where *duration2* is the time between their Home Visit and their Clinical Exam (i.e., time between HbA1c_initial_ and HbA1c_final_). Although the covered time period was relatively short, we included these durations in this analysis to account for secular increases in HbA1c with age and to reduce any other effect that temporal variation has on results. The parameter of interest is b1, which corresponds to an association with individual chemicals (traditional Tobit regression) or an association with the BTEX mixture (Tobit QGcomp).

**Table 1:** Exposure distributions of cumulative sum of daily maximum exposures for analyses in parts per billion-days

	Minimum	Quartile 1	Median	Quartile 3	99th percentile	Maximum
Benzene	0.04	150.75	498.76	1257.73	5725.05	10,591.67
Toluene	0.24	574.96	2106.54	4828.88	17,361.21	29,656.55
Ethylbenzene	0.02	122.62	364.58	876.62	3287.96	4596.40
Xylenes	2.35	1065.12	2466.10	4970.30	14,917.00	19,918.85
Total BTEX	6.24	1919.34	5361.89	11,300.47	33,822.48	56,337.23

We addressed covariate data missingness using multiple imputation with chained equations (MICE) [62, 63] (see Table 2 for missingness in relevant variables), creating 14 imputed datasets. This analysis includes some individuals who were taking diabetes medication at the time of the Home Visit or who started diabetes medication during follow-up. To account for the impact of this medication use on the study outcomes, we used a Tobit regression [64, 65] paired with censoring weights (where “censored” refers to taking diabetes medication). Tobit regression is a censored linear regression– in this case the untreated (i.e., “true”) outcome value (either initial HbA1c or final HbA1c) is assumed to be as high or higher than the treated (i.e., observed) value. For those not taking diabetes medication, untreated values were equal to their observed values. Because Tobit regression accounts for potential censoring only in the outcome, in analyses of final HbA1c in Eq. 3. we artificially censored taking HbA1c-lowering medication at the initial HbA1c and used inverse probability of censoring weights to account for individuals taking HbA1c-lowering medication at the initial HbA1c measurement. For analysis of final HbA1c the weights also accounted for probability of HbA1c-lowering medication use at the time of the Clinical Exam to account for additional factors (outside of the analytical Tobit regression model) related to likelihood of this medication use. We constructed inverse probability weights for treatment at the Home Visit and Clinical Exam, separately, using logistic regression models that included all variables that might affect a participant’s treatment status as well as interactions between relevant variables (see Supplementary Table 1). We stabilized these weights with variables included in the analysis models (age at Home Visit and BMI). Weights for treatment at the Home Visit applied to all analyses, and the weights for treatment at the Clinical Exam were additionally applied to models examining HbA1c at the Clinical Exam. We stabilized these weights with variables included in the analysis models (age at Home Visit and BMI). We used Rubin’s rules to aggregate results across 14 imputed datasets [66]. For QGcomp models, we assumed linearity and additivity amongst BTEX exposures, which allows for a computationally efficient procedure in which coefficients from a Tobit model that includes all exposures and confounders is fit to “quantized” exposures and the coefficients from the exposures are summed to estimate the overall effect of the BTEX mixture.

**Table 2:** Descriptive characteristics of the study sample compared to the overall cohort

		Gulf Study workers	Analysis sample
		*N* = 24,438	*N* = 2541
Age^a^ (years) (mean (SD))		43.95 (12.9)	45.76 (12.9)
	Missing	-	-
Race (%)			
	Black	5781 (23.7)	1015 (39.9)
	Other	2441 (10.0)	199 (7.8)
	White	16,215 (66.4)	1327 (52.2)
	Missing	1	0
Sex (%)			
	Male	20,135 (82.4)	1979 (77.9)
	Female	4303 (17.6)	562 (22.1)
	Missing	-	-
Educational attainment (%)			
	Less than HS	3840 (15.8)	554 (21.8)
	HS/GED	7181 (29.5)	862 (33.9)
	Some college	7319 (30.0)	781 (30.7)
	4 year college/more	6030 (24.7)	344 (13.5)
	Missing	68	0
Employment^b^ (%)			
	Other^f^	2158 (8.9)	372 (14.7)
	Unemployed	4988 (20.5)	749 (29.5)
	Working/student/keeping house	17,191 (70.6)	1414 (55.8)
	Missing	101	6
Smoking status^b^ (%)			
	Former smoker	5175 (21.5)	562 (22.4)
	Heavy current smoker	2433 (10.1)	277 (11.0)
	Light current smoker	4767 (19.8)	570 (22.7)
	Never smoker	11,730 (48.7)	1098 (43.8)
	Missing	333	34
Potential prior exposure to BTEX-H^c^ (%)			
	No	9403 (38.5)	963 (37.9)
	Yes	15,033 (61.5)	1576 (62.1)
	Missing	2	2
Body Mass Index^a^ (kg/m^2^) (mean (SD))		29.80 (6.64)	30.00 (6.48)
	< 25	2144 (24.1)	582 (23.1)
	25–30	2964 (33.3)	821 (32.6)
	≥ 30	3790 (42.6)	1119 (44.4)
	Missing	15,540^e^	19
ADI^d^ quartile (%)			
	1/2	9603 (40.6)	721 (28.5)
	3	7472 (31.6)	888 (35.1)
	4	6557 (27.7)	921 (36.4)
	Missing	806	11
Health insurance status^a^			
	No	4486 (50.9)	1260 (50.3)
	Yes	4328 (49.1)	1246 (49.7)
	Missing	15,624^e^	35
All data were from the Gulf Long Term Follow-up Study of individuals who worked for at least one day on the *Deepwater Horizon* oil-spill cleanup and who completed a routine clinical exam as part of that study 4–6 years after the spill. a At the Home Visit (2011–2013) b At Enrollment (2011–2013) c Potential previous occupational exposure for at least 30 min a week to benzene, toluene, ethylbenzene, xylenes, or n-hexane d At Enrollment residence, Area Deprivation Index in 2013 e Many were not able to provide information at the Home Visit due geographic eligibility f Employment Other category includes disabled (permanently or temporarily), retired, and other			

We present fully adjusted analyses of initial HbA1c and of final HbA1c not adjusting for initial HbA1c. We also present fully adjusted analyses and sub-analyses for final HbA1c adjusting for initial HbA1c. We determined covariates using a directed acyclic graph [67] (see Supplementary Fig. 2). Covariates considered included, at the time of the Home Visit, age (months), sex (male/female), race (Black, White, Other), highest educational attainment (less than high school, high school/GED, some college, 4 year college degree or more), employment status (working/student/keeping house, unemployed, other), smoking status (former smoker, current light smoker, current heavy smoker, never smoker), health insurance status (yes/no), measured body mass index (BMI) (kg/m^2^), potential previous occupational exposure to BTEX for at least 30 min per week (yes/no), quartiles of nationally scaled Area Deprivation Index at Enrollment residence (ADI quartile [Q]1/2, Q2, Q3, Q4), and physical activity (minutes per week of moderate or vigorous physical activity). We include race as a proxy for lived experience not captured by the other covariates. The ADI is a previously validated measure of area level socioeconomic status and is derived from US census variables; higher values are associated with relatively more deprivation compared to the rest of the US population [68–70]. We completed a functional form analysis for all continuous covariates, comparing bivariate models of the outcome with each covariate coded as linear, squared, natural splines with 3 knots, and natural splines with 5 knots. We selected the best coding scheme using the Akaike Information Criterion. For all covariates except physical activity, the continuous linear coding was the best, or tied for best, fit. Physical activity did not contribute to the model fit, so we did not include it in the final model. We assessed differences in the relationship between exposures and final HbA1c (controlling for initial HBA1c). Because benzene, ethylbenzene, and xylenes are accumulated in fat tissue [71, 72], as is toluene [73], we stratified by BMI categories (underweight/normal weight: <25 kg/m^2^, overweight: 25–29.9 kg/m^2^, obese: ≥30 kg/m^2^). Because the burden of DM differs by race and socioeconomic position, we stratified, separately, by race (Black or White) and ADI (Q1/2, Q3, Q4).

To ensure that patterns of effects were not due to categorizing the exposures into quartiles or weighting, we performed adjusted weighted and un-weighted Tobit regressions (see Supplementary Fig. 3 and Supplementary Fig. 4), using splines to represent the exposures (5 knots) and all continuous covariates (3 knots). See Supplementary Fig. 5 for results from unweighted linear regression for HbA1c_final_ controlling for HbA1c_initial_ including all participants (direct comparison to Fig. 3).

We completed all analyses using R [74](version 4.2.1) packages included in the *tidyverse* [75] as well as individual packages *mice* [76], *qgcomp* [77], *ipw* [78], and *AER* [79].

## Results

Compared to the overall cohort of Gulf Study workers (Table 2), those included in this analysis were slightly older at the Home Visit (46 vs. 44 years), less likely to self-identify as White (52% vs. 66%), more likely to be female (22% vs. 18%), less likely to have completed a 4 year college degree or more (14% vs. 25%), less likely to be full-time working/studying/keeping house (56% vs. 71%), slightly less likely to have never smoked (44% vs. 49%), and less likely to live in ADI Q1/2 (least deprived) areas (29% vs. 41%). This study sample was comparable to all workers who participated in the Home Visit in BMI, potential prior occupational exposure to BTEX, and health insurance status at the Home Visit. For descriptive information by quartile of cumulative total BTEX, see Supplementary Table 8.

### Home visit HbA1c

**Fig. 2:**
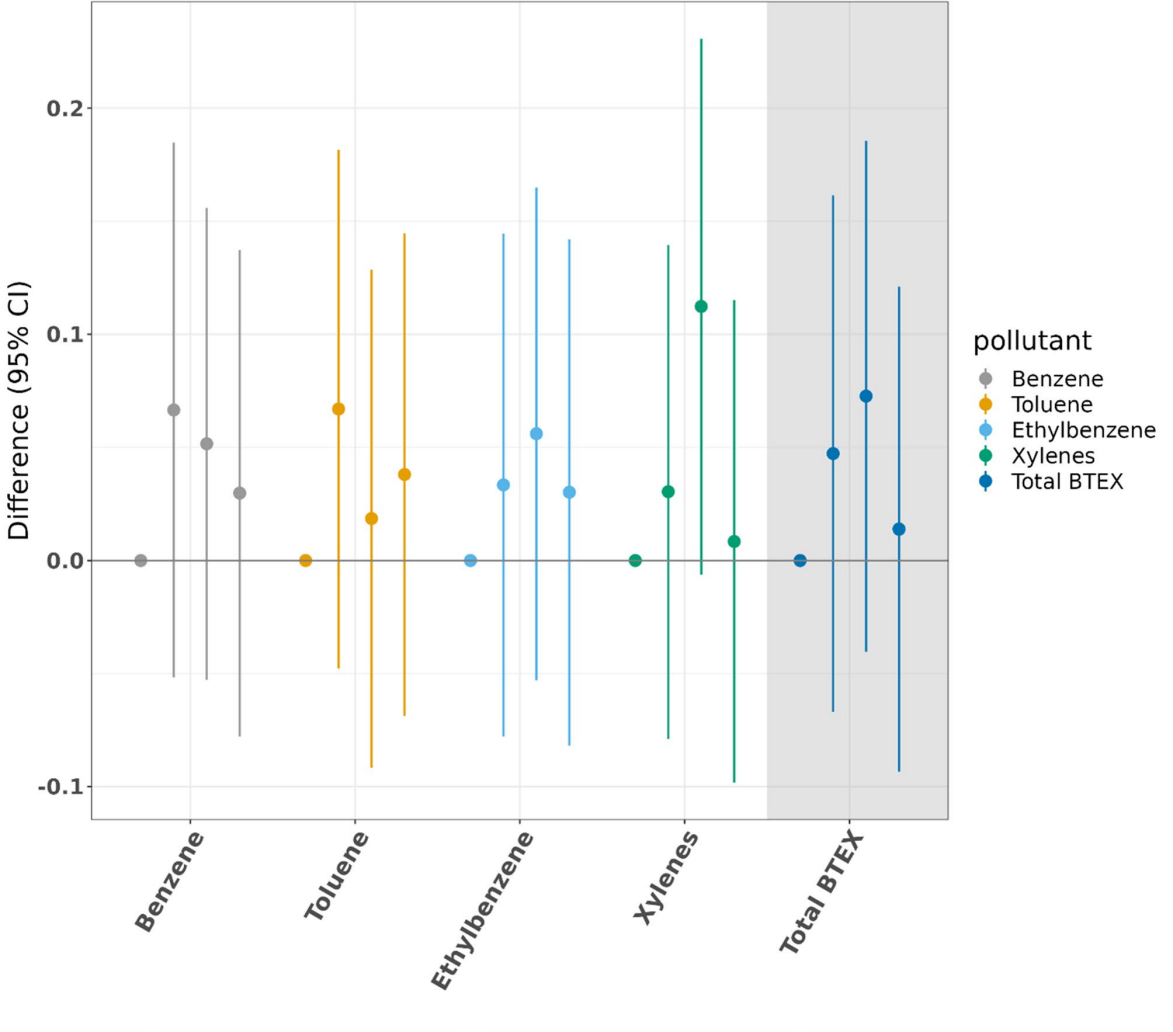
Fully adjusted results (estimates and 95% confidence intervals) examining mean difference in home visit (initial) HbA1c (%) by quartile of cumulative exposure when compared to Q1. For each of the BTEX exposures, there are four point estimates, representing, from left to right, Q1 (reference, always 0) and then Q2, Q3, and Q4 (with lines denoting 95% confidence intervals) compared with Q1. Each pollutant is given a different color for ease of differentiation

We observed no clear patterns of association between any of the individual BTEX chemicals, total BTEX, and HbA1c level at the Home Visit, although the upper quartiles (Q2 or Q3) of exposure were associated with considerable overlap and non-significantly elevated HbA1c compared to the reference quartile for each pollutant in adjusted analyses (Fig. 2, Supplementary Table 2). The QGcomp result showed that a 3-quartile increased exposure to the mixture was associated with non-statistically higher values of initial HbA1c compared to Q1, though with a wide confidence interval (0.13, 95%CI (-0.16, 0.43)).

### Final HbA1c

#### Adjusted

**Fig. 3:**
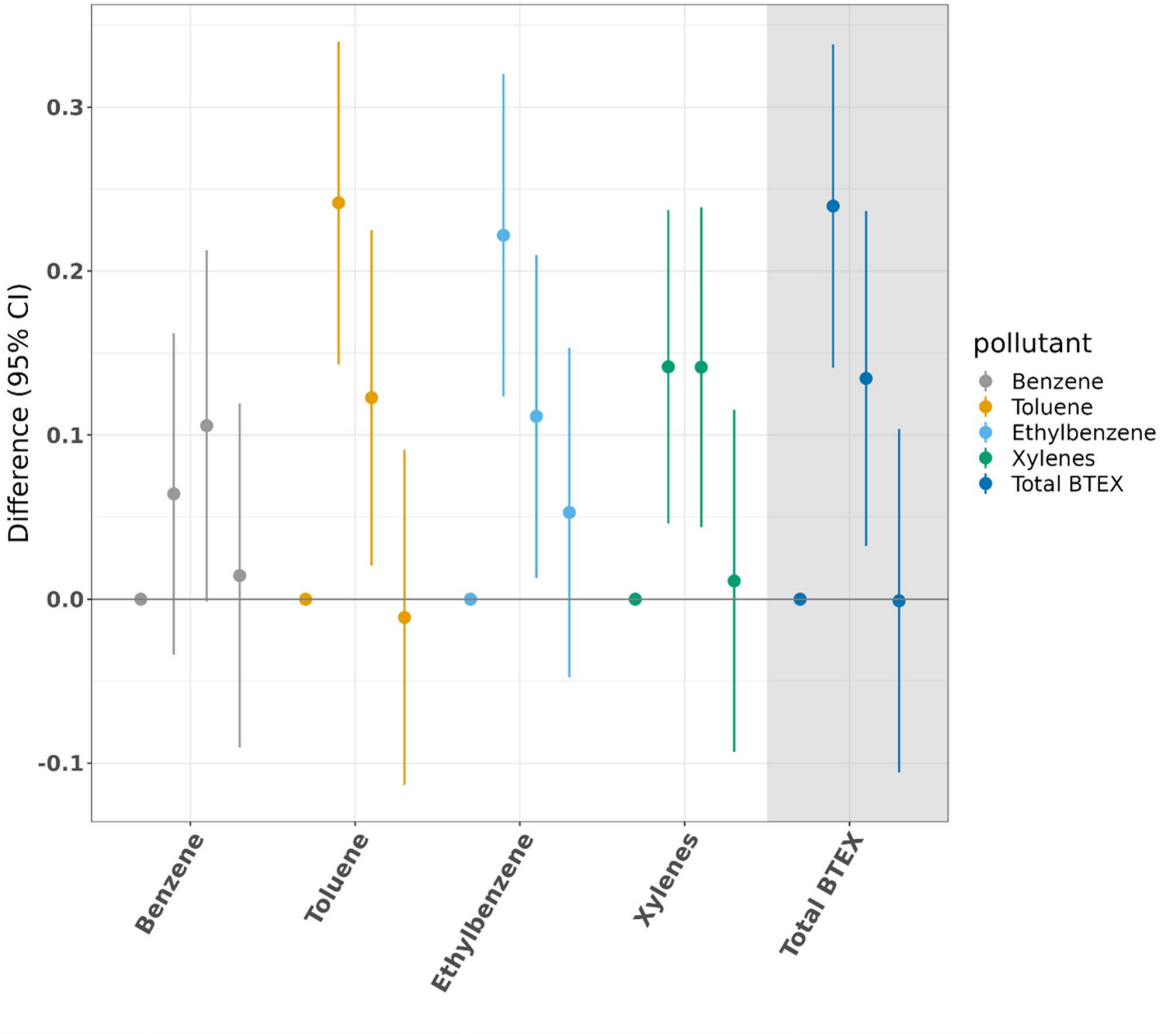
Fully adjusted results (estimates and 95% confidence intervals) examining mean difference in clinical exam (final) HbA1c (%) by quartile of cumulative exposure when compared to Q1, controlling for home visit (initial) HbA1c. For each of the BTEX exposures, there are four point estimates, representing, from left to right, Q1 (reference, always 0) and then Q2, Q3, and Q4 (with lines denoting 95% confidence intervals) compared with Q1. Each pollutant is given a different color for ease of differentiation

Age-adjusted results are available in Supplementary Table 3. In fully adjusted models of final HbA1c adjusting for initial HbA1c, we observed that Q2 and Q3 exposures generally were associated with a statistically significant higher final HbA1c compared to Q1 (Fig. 3, Supplementary Table 4), although with substantial overlap. As reflected in the QGComp estimate of the joint association for a 3-quartile increase in each BTEX component (-0.06, 95% CI (-0.14, 0.03)), there were generally decreasing HbA1c levels with increasing exposure from Q2 to Q4.

The results from fully adjusted models for final HbA1c not adjusting for initial HbA1c have a similar pattern of Q2 or Q3 showing the highest magnitude positive estimates and Q4 either null or below the null (Supplementary Fig. 6).

The results of the unweighted linear regression (Supplementary Fig. 5), performed for comparison to Fig. 2, show a similar inverted-U pattern whereby Q2 is consistently associated with the highest HbA1c difference compared to Q1 for the BTEX chemicals and for total BTEX. These results are known to be biased due to the lack of weighting, but the continued presence of the inverted-U pattern indicates that this shape is not due to the weighting.

#### Stratified body mass index analyses

**Fig. 4:**
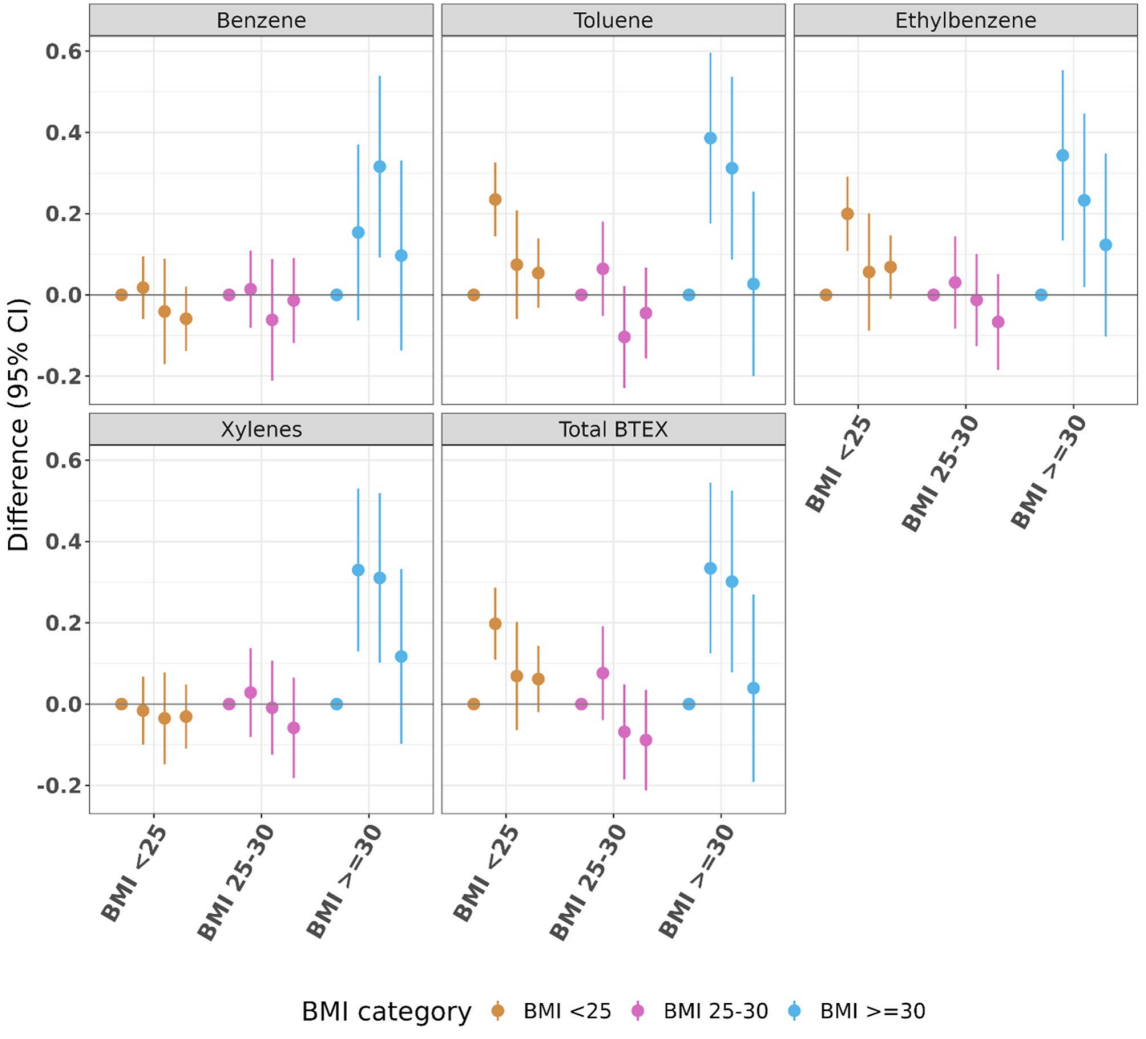
Fully adjusted results (estimates and 95% confidence intervals) examining mean difference in clinical exam (final) HbA1c (%) by quartile of cumulative exposure when compared to Q1, stratified by body mass index category and controlling for home visit (initial) HbA1c. For each of the BTEX exposures, there are four point estimates, representing, from left to right, Q1 (reference, always 0) and then Q2, Q3, and Q4 (with lines denoting 95% confidence intervals) compared with Q1. Each pollutant is given a different color for ease of differentiation

In both the strata with normal/underweight and overweight individuals, we observed no discernable patterns (Fig. 4, Supplementary Table 5). The mixture estimate for a simultaneous 3-quartile increase in each BTEX chemical given by QGcomp for under/normal weight individuals was null (-0.02, 95% CI (-0.09, 0.06)) as was the estimate for overweight individuals (-0.04, 95% CI (-0.14, 0.07)).

Among obese participants (BMI > 30) we observed estimates that were more positive than those from the other BMI strata and patterns generally were similar to those seen in the fully adjusted analysis of final HbA1c adjusting for initial HbA1c. For all pollutants, Q2 and Q3 exposures were associated with higher final HbA1c values compared to Q1, and again Q4 estimates were closer to null, which demonstrated an inverse “U” shaped association. The estimate for a 3-quartile increase in each mixture component given by QGcomp was also null (-0.07, 95% CI (-0.26, 0.12)).

#### Stratified area deprivation index

**Fig. 5:**
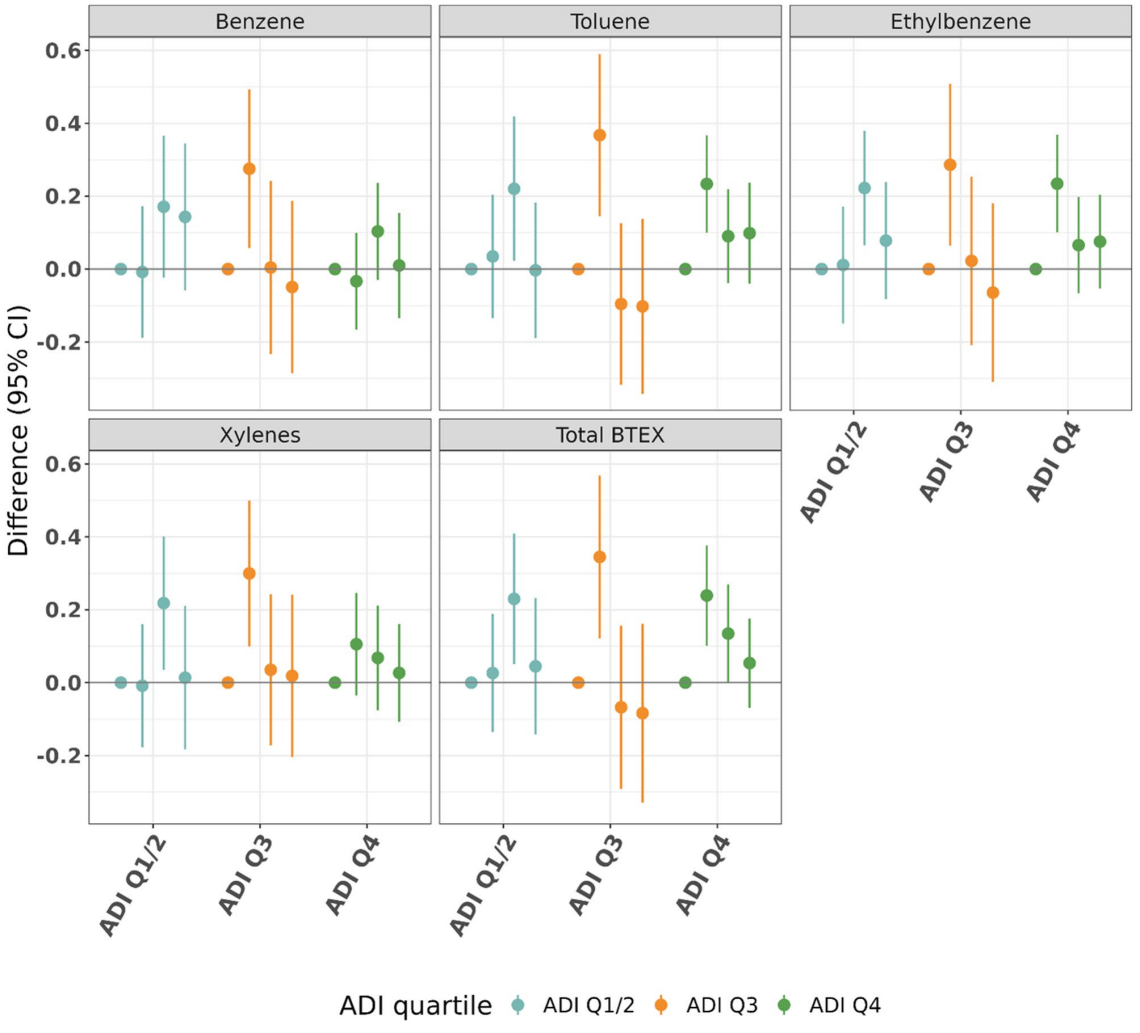
Fully adjusted results (estimates and 95% confidence intervals) examining mean difference in clinical exam (final) HbA1c (%) by quartile of cumulative exposure when compared to Q1, stratified by quartile of area deprivation index and controlling for home visit (initial) HbA1c. For each of the BTEX exposures, there are four point estimates, representing, from left to right, Q1 (reference, always 0) and then Q2, Q3, and Q4 (with lines denoting 95% confidence intervals) compared with Q1. Each pollutant is given a different color for ease of differentiation

There were few discernable differences in patterns of ADI quartile-stratified results, and estimates had considerable confidence interval overlap between strata (Fig. 5, Supplementary Table 6). In results among those in the least deprived ADI category (ADI Q1/2), we observed in all pollutants that Q3 exposures were associated with the highest final HbA1c values compared to Q1. In results among those in the ADI Q3 stratum we observed that for all pollutants Q2 exposure was associated with the highest final HbA1c value in relation to Q1, with other estimates null or slightly below the null. In results among those in the ADI Q4 stratum exposure Q2 estimates were generally the most elevated compared to Q1.

The estimate for a 3-quartile increase in mixture components given by QGcomp for both the ADI Q1/2 and ADI Q4 strata were null (ADI Q1/2 0.00, 95% CI (-0.16, 0.16); ADI Q4 0.04, 95% CI (-0.08, 0.16)). The mixture estimate given for QGcomp for the ADI Q3 stratum was markedly associated with lower final HbA1c values (-0.18 (-0.37, 0.00)).

#### Stratified by race

**Fig. 6:**
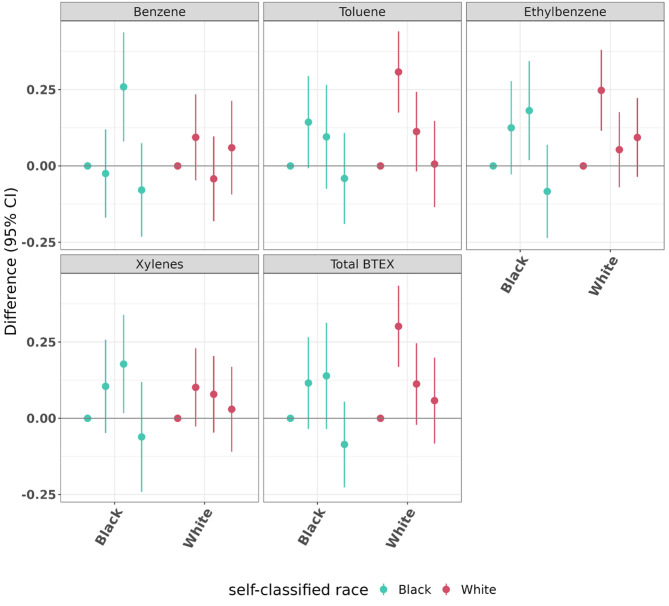
Fully adjusted results (estimates and 95% confidence intervals) examining mean difference in clinical exam (final) HbA1c (%) by quartile of cumulative exposure when compared to Q1, stratified by self-classified race and controlling for home visit (initial) HbA1c. For each of the BTEX exposures, there are four point estimates, representing, from left to right, Q1 (reference, always 0) and then Q2, Q3, and Q4 (with lines denoting 95% confidence intervals) compared with Q1. Each pollutant is given a different color for ease of differentiation

In race-stratified results, we did not observe major differences in the patterns of results, and pollutant estimates had considerable overlap in confidence intervals between strata (Fig. 6, Supplementary Table 7). In results among those who self-classified as Black we observed that Q2 or Q3 exposures were associated with the highest final HbA1c values (adjusted for initial HbA1c) relative to Q1 for most pollutants and Q4 estimates were null or negative. In results from those who self-classified as White we observed that Q2 exposures were associated with the highest final HbA1c values relative to Q1. The mixture estimates given by QGcomp for both strata were null (Black − 0.05, 95% CI (-0.19, 0.09); White − 0.04, 95% CI (-0.15, 0.08)).

## Discussion

This analysis examined associations between oil spill cleanup-related exposures and latent, untreated HbA1c, both 1–3 years after exposure and 4–6 years after exposure. Exposure to the aggregated sum of BTEX was suggestively positively associated with latent, untreated *initial* HbA1c, but the relationship was more complicated with latent, untreated *final* HbA1c 3 years later. We did not observe monotonic trends in associations of individual BTEX pollutants or of total BTEX with latent, untreated final HbA1c, instead observing an inverted-U pattern with elevations in the second and third quartiles, but not the fourth quartile, compared to the first quartile for the majority of analyses. Most of the point estimates for Q2 and Q3 were above the null in these associations and overlapped other quartiles in the same pollutant analysis. Patterns of association varied little from these findings when we stratified the analysis by BMI category, by ADI quartile, or by race. The analysis sample included higher proportions of individuals who are at higher risk of developing diabetes (i.e., those who self-identify as Black, those who have lower educational attainment, those who live in more deprived areas). While this analysis adjusted for these factors and therefore the pattern of estimates would be similar in the overall GuLF Study worker population, the distribution of the outcome may be slightly different in the overall GuLF Study worker population.

In this analysis we found inverted-U patterns (whereby Q2 and/or Q3 are most elevated when compared to Q1) which, while there are not yet established explanations as to why this would be, have been found before in a related study. Choi et al. [5]. examined a urinary benzene metabolite and insulin resistance over a 2-year period and found that increased levels of the metabolite were associated with elevated levels of insulin resistance with the same inverted-U shape shown in the present study. As discussed in a review by Bolden et al. (2015) [80] review, the BTEX chemicals show evidence of disrupting the human endocrine system, through disruption of specific hormones and related health outcomes. Non-monotonic dose-response patterns have been seen in relation to other endocrine disrupting chemicals [81, 82]. Additionally, it is known that diabetogenic responses to endocrine disruptors can be non-monotonic [83]. It is also possible that this pattern is due to chance. This pattern was unlikely produced by unmeasured confounding, though, as we were able to control for many confounders. The persistence of the inverted-U shape in these analyses and its presence in previous literature indicate that this finding should be further investigated.

Results from the aggregate total BTEX and the QGComp mixture analysis provide valuable insights. The results from total BTEX have similar magnitudes to those of the individual BTEX chemicals. This indicates that exposure effects were not all additive: if all of the associations were additive, we would expect to see very different magnitudes of results for total BTEX when compared to the individual BTEX results. Additionally, the mixture estimate, strongly negative only in the ADI Q3 stratum and null at all other times, even in situations when the point estimates for individual BTEX and total BTEX were all above the null, is consistent with the general patterns whereby Q2 or Q3 was most elevated and Q4 was null or negative.

Differences seen in the BMI-stratified analysis may reflect a difference in pollutant metabolism or physiological response mechanisms. The more positive estimates seen among obese participants may indicate that these individuals have physiological compensation mechanisms that are less efficient (greater physiological response, slower rate of recovery, or less overall capacity to compensate) than those who are not obese. These results may also be related to literature showing that benzene, ethylbenzene, xylenes [72], and toluene [73] preferentially accumulate in fatty tissue. However, the numbers in stratified analyses are small, and it is not possible to conclude that the effects are truly different among obese versus not obese participants. Chemicals and metabolites that are deposited in adipose tissue are not eliminated as quickly from the body [72]. Therefore, those with a greater amount of adipose tissue may experience a longer overall exposure duration after chemical inhalation. Prolonged body burden of the chemicals or their metabolites may convey greater risk of physiologic damage than shorter body burden (i.e., with faster elimination from the body).

Results from the ADI-stratified analysis and race-stratified analysis did not show clear stratum-specific patterns. We expected estimates for those who self-identified as Black or those who lived in more deprived areas to show larger magnitude difference estimates due to increased allostatic load and thus decreased capacity to compensate for physiological damage and increased susceptibility to glycemic dysfunction from chemical exposure. Indeed, increased allostatic load has been associated with metabolic dysfunction previously [84]. It is possible that the covariates and stratification variables available for this analysis were not sufficiently specific to capture the stratum-related factors that contribute to allostatic load, in contrast to biomarkers commonly used for gauging allostatic load, including, but not limited to, cortisol, epinephrine, norepinephrine, and dehydroepiandrosterone sulfate [85]. Additionally, there is literature showing that those who are most socioeconomically disadvantaged experience higher levels of environmental exposures [86–88], thus possibly confounding analyses of occupational exposures sustained during OSRC. That is, individuals with high allostatic load and relevant non-occupational environmental exposures may have already experienced and incompletely recovered from physiological damage leading to altered compensation mechanisms or glycemic regulation.

There has been limited research of the relationship between the BTEX chemicals and HbA1c level or diabetes risk. Besides the previously mentioned study of insulin resistance that found a similar inverted-U pattern, there have been a few others. Cross-sectional analyses suggest that increased levels of exposure to benzene, ethylbenzene, and xylenes (analyzed separately) are associated with higher HbA1c in the general Canadian population [4]. We had reason to believe that this analysis would show some positive associations: higher benzene metabolite levels in urine have been cross-sectionally linked to higher levels of insulin resistance (which leads to higher HbA1c) in children and adolescents [3]. Finally, rodent studies indicate that adverse physiological response can occur relatively quickly following exposure: immediately following inhaled exposure to benzene 6 h a day for 2 weeks, mice showed indications of glycemic dysregulation (higher fasting blood glucose and insulin) [89].

Selection into the analysis is unlikely to have induced the inverted-U shape, and specifically the low Q4 point estimates. Those in Q4 were no more likely to have died or otherwise been lost to followup than those in the other exposure categories (see Supplementary Table 9). Additionally, it is unlikely that migration out of the Clinical Exam catchment areas to seek treatment for severe illness would be related to elevated HbA1c levels: only 8% of worker participants in the Gulf states moved counties between enrollment and June 2016, and the Clinical Exam catchment areas encompassed metropolitan areas with medical systems capable of managing elevated HbA1c.

Additionally, procedures for calculating exposure estimates for work groups and calculating each worker’s sum of daily maximum exposures over their work duration were unlikely to have altered the pattern of results. These exposure estimates for the GuLF Study have been well received by the field and used in many other publications despite challenges presented by estimating exposures. Though there was 60% occurrence of below-detection readings for individual chemicals, GuLF Study industrial hygienists were able to estimate individual BTEX exposures from other chemical readings, that were detectible, using Bayesian methods. Accepted exposure estimates were required to have an average relative bias of < 15% and an average relative rMSE of < 65%. This bias and precision is about the same or somewhat less than the bias and precision expected by the actual measurement methods for each of the 6 chemicals (THC and BTEX-H). Therefore, the estimation methods used to develop the exposure estimates are unlikely to have had a large impact on the risk estimates. Additionally, calculating each worker’s sum of daily maximum estimated exposures is unlikely to have induced the pattern of point estimates, as the criteria for exposure estimate acceptance was not different between jobs with high and low exposures. Errors in exposure estimates would have reduced precision of the observed point estimates without inducing a specific pattern. However, procedures for calculating exposure estimates resulted in estimates with error comparable to that of measured exposures.This analysis has several limitations. One limitation is that both HbA1c measures were collected after exposure. Such a limitation is unavoidable for most studies of the health effects of disasters. This means that we were not able to examine differences in HbA1c levels between the pre- and post-exposure periods. However, our results can indicate differences in trajectory of HbA1c following exposure as we were able to examine HbA1c at two separate time points post-exposure. Secondly, this analysis addresses associations of exposures with latent, untreated HbA1c (that is, untreated HbA1c) using Tobit regression. This modeling strategy allows *estimation* of the unobservable untreated value in a counterfactual (non-existent) world in which nobody is treated with HbA1c-lowering medication. It allowed us to not assume that the outcome is fixed and known - this is reflected in potentially wider confidence intervals for our point estimates than we would have if we treated the outcome as known. By considering HbA1c as a censored variable, we are potentially sensitive to bias from informative censoring by factors not accounted for in the model [90]. To address this concern, we included a range of predictors of HbA1c level in our Tobit models and associated weights to best estimate these values (see Methods section). Additionally, inclusion in the analytic model of a baseline HbA1c measurement when examining a later measurement of HbA1c creates the possibility for artifactual signals due to regression to the mean arising from measurement error [91], though use of the above model form, where the initial HbA1c is a covariate instead of being incorporated into an outcome of change, greatly reduces this possibility. Also, analyses of final HbA1c without adjusting for initial HbA1c showed the same patterns, indicating that any regression to the mean did not greatly impact result patterns. Additionally, we used exposure estimates derived from a JEM in these analyses. These exposure estimates are more susceptible to error than are individual-level exposure measurements (e.g., from a dosimeter) due to variation in exposure within job-groups. Representing exposures as quartiles decreases the probability that an individual has error in the exposure measure being used in analysis. It is not clear how any remaining exposure misclassification might affect results. This misclassification is non-differential with respect to the outcome, which generally (but not always) leads to estimates moving closer to the null [89]. Use of JEMs is common in occupational epidemiology studies. Moreover, these exposure estimates are more refined than those used in other studies examining the health of individuals exposed to oil spills, which have mostly categorized individuals as exposed/unexposed or simply by number of days exposed. The JEM incorporates measurements taken at different time points over the response and clean-up experience whereas it would not have been feasible to obtain multiple exposure measurements for all participants throughout the spill, even if we had been able to capture them in real time versus after most of the clean-up had ended. While it has good internal validity due to use of multiple imputation to address covariate missingness, it may not be directly generalizable to all other populations. Populations for which the distributions of key covariates differ (e.g., much younger age) may have different factors that are important to consider and, therefore, the comparability of other populations should be determined prior to generalizing.

Despite these limitations, this study has marked strengths. This is the first study to examine HbA1c among oil-spill cleanup workers. It uses a well-powered subsample from the largest and longest followed-up cohort of oil-spill workers with detailed exposure data. It also has extensive demographic, behavioral, and anthropometric data, which allowed us to account for many potential confounders and effect measure modifiers. Multiple imputation allowed us to retain participants who were missing covariate information. As missingness was very low (highest % missingness in any variable was 35/2541 * 100% = 1.4% of the analysis sample), multiple imputation allowed us to achieve greater precision with low likelihood of notably changing point estimates from what they would have been if the analysis sample had had complete information. The sample is large enough that we were able to stratify by key demographic and anthropometric factors, thus allowing us to investigate associations among potentially more sensitive subgroups [92]. Additionally, the exposure estimates used in this study are based on the most rigorous exposure reconstruction ever performed for an oil spill-exposed population. Finally, there is unlikely to be substantial measurement error in either HbA1c value — both values were measured with techniques approved by the National Glycohemoglobin Standardization Program for HbA1c measurement and agreement between replicate samples was very high.

The results of this study suggest that exposures to the moderate levels of exposure to the BTEX chemicals observed in this study population, individually and as an aggregate, may be associated with elevated HbA1c up to 6 years after exposure, with an inverted U-pattern. There are no indications of monotonic trends between exposure to these chemicals and HbA1c and there was substantial overlap among the estimates. More research is needed to identify the physiological mechanisms that may have contributed to the observed associations. Such research would be strengthened by measurement of HbA1c prior to exposure and at regular time points post-exposure.

## Electronic supplementary material

Below is the link to the electronic supplementary material.

Supplementary Material 1

## Data Availability

The GuLF Study data is available upon research proposal approval. Find more information at https://gulfstudy.nih.gov/en/forresearchers.html.
