# Supplementary material for: Prospective study of oil spill cleanup-related exposure to volatile organic compounds and glycemic dysregulation
Source: Environ Health. 2025 Sep 26;24:67. doi: 10.1186/s12940-025-01211-5 (PMC12465997; doi:10.1186/s12940-025-01211-5)
Supplement: Supplementary file 1 — Supplementary Material 1 [file 12940_2025_1211_MOESM1_ESM.docx]

# Supplement for: Prospective study of oil spill cleanup-related exposure to volatile organic compounds and glycemic dysregulation


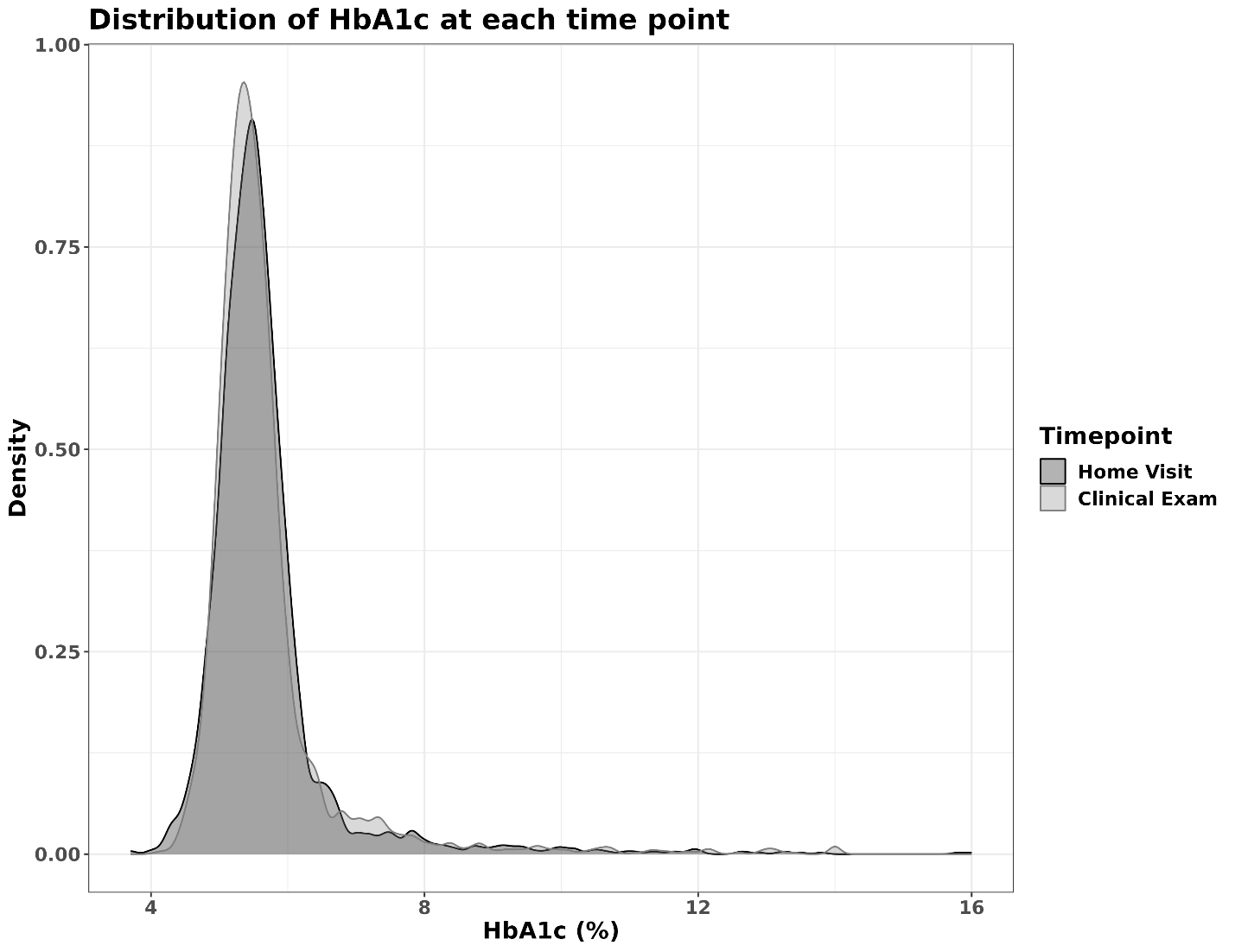


Supplementary Figure 1: Distribution of HbA1c (%) at the Home Visit and Clinical Exam


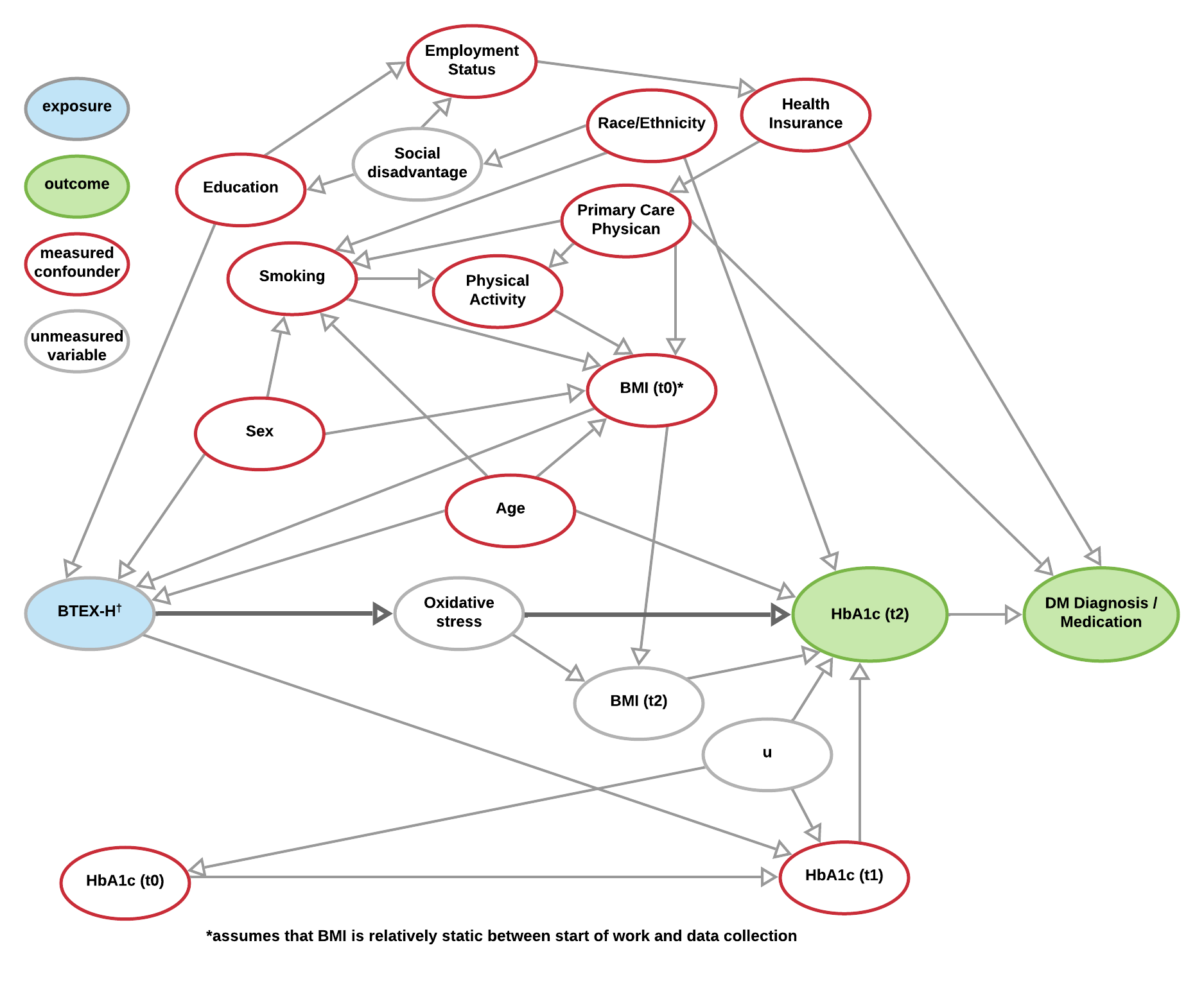


Supplementary Figure 2: Directed Acyclic Graph (DAG) for this analysis


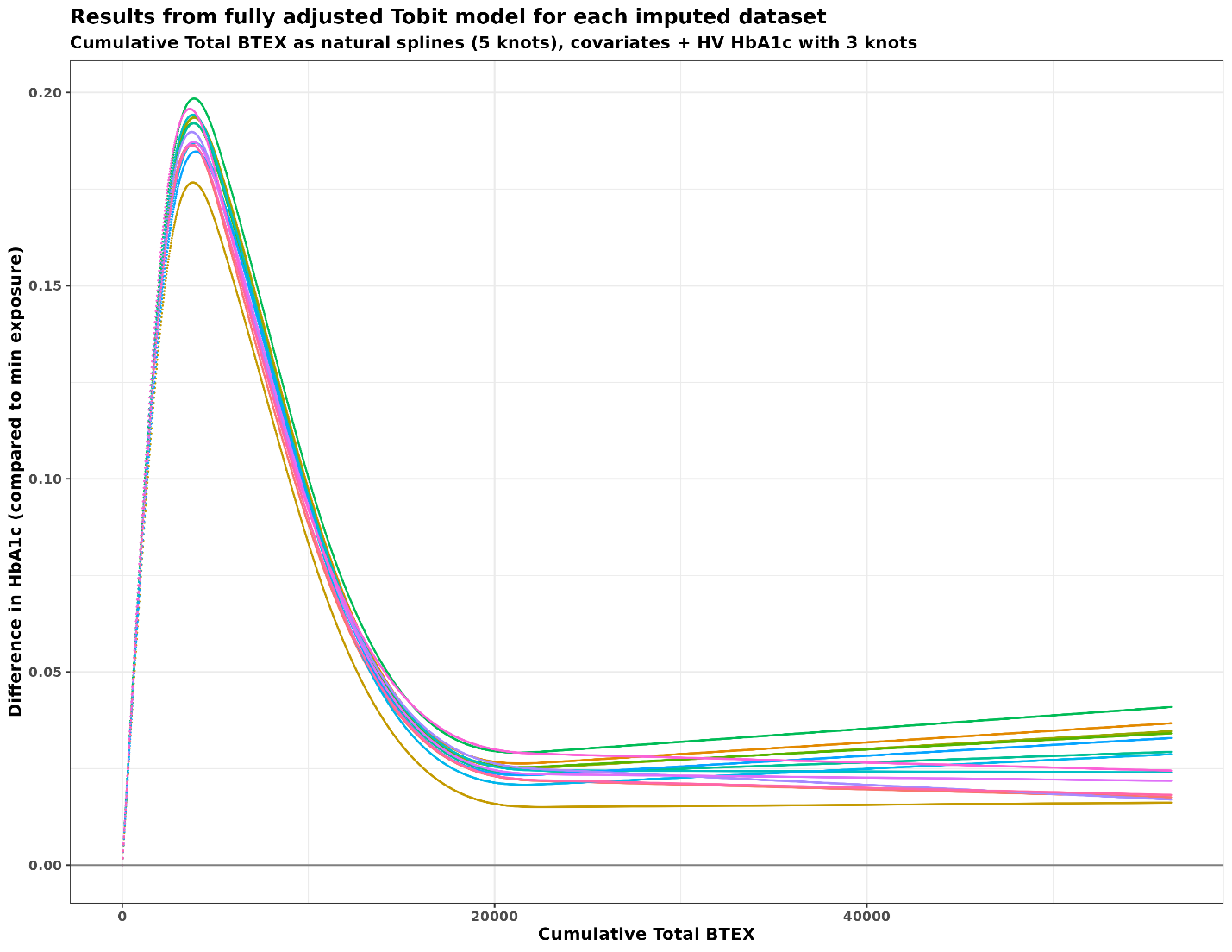


Supplementary Figure 3: Results from fully adjusted, weighted, Tobit regression comparing predicted HbA1c to predicted HbA1c at the minimum exposure. Total BTEX was modeled with natural splines (5 knots), Home Visit HbA1c with natural spline (3 knots), and all continuous covariates with natural splines (3 knots). Each line represents results from one imputed dataset


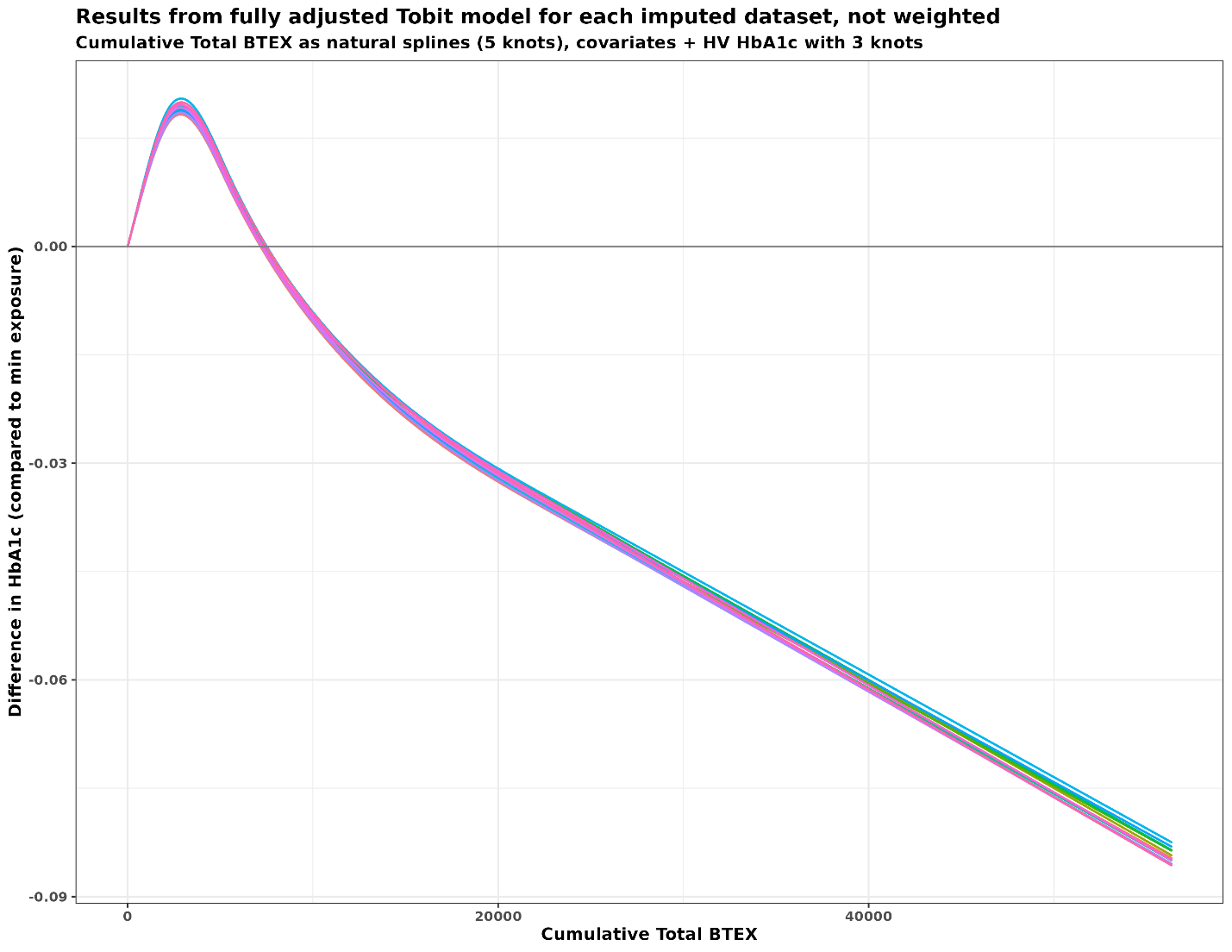


Supplementary Figure 4: Results from fully adjusted, unweighted, Tobit regression comparing predicted HbA1c to predicted HbA1c at the minimum exposure. Total BTEX was modeled with natural splines (5 knots), Home Visit HbA1c with natural spline (3 knots), and all continuous covariates with natural splines (3 knots). Each line represents results from one imputed dataset.


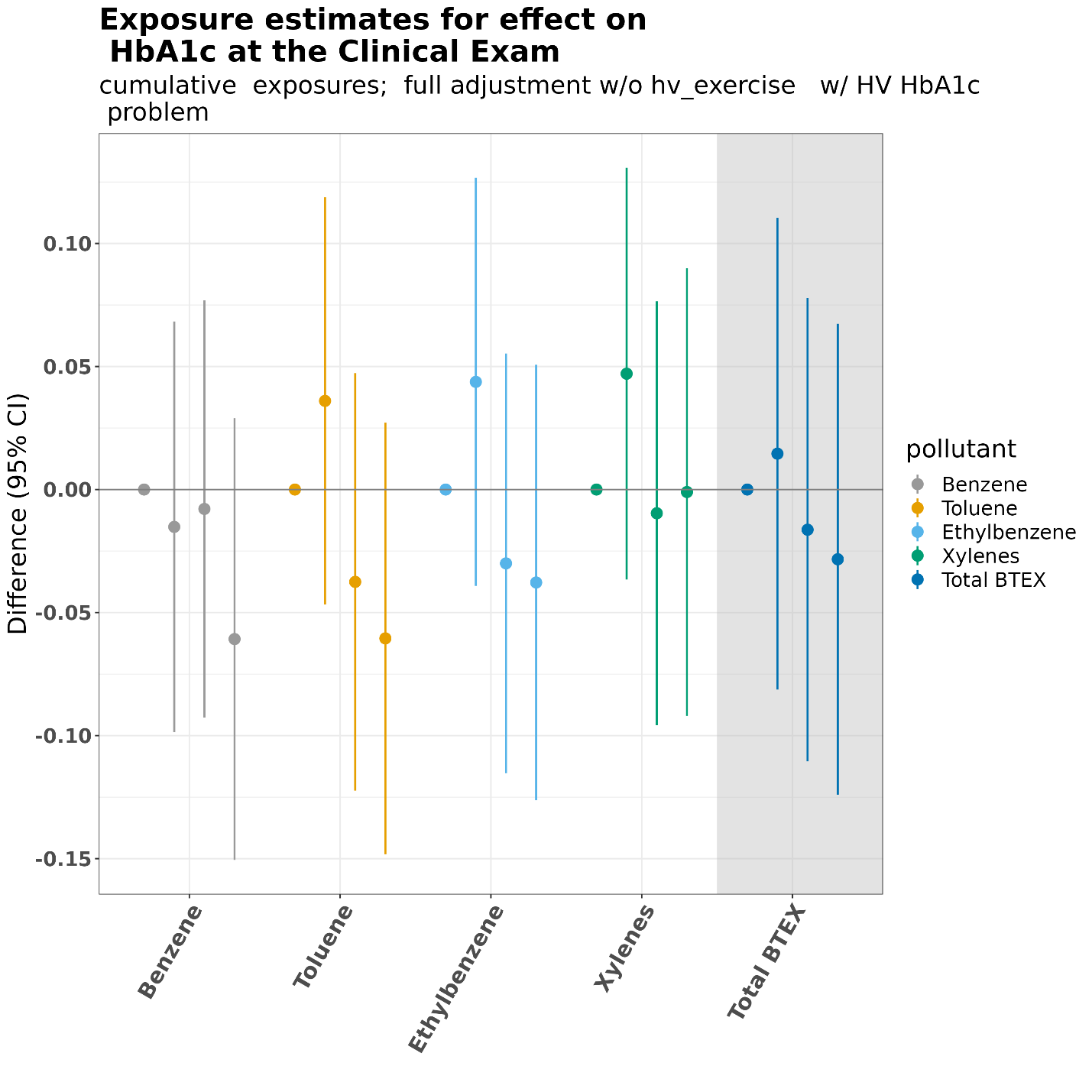


Supplementary Figure 5: Results from fully adjusted unweighted linear regression examining mean difference in HbA1c (%) at the Clinical Exam by quartile of cumulative exposure when compared to Q1, controlling for Home Visit HbA1c. For comparison to Tobit regression result


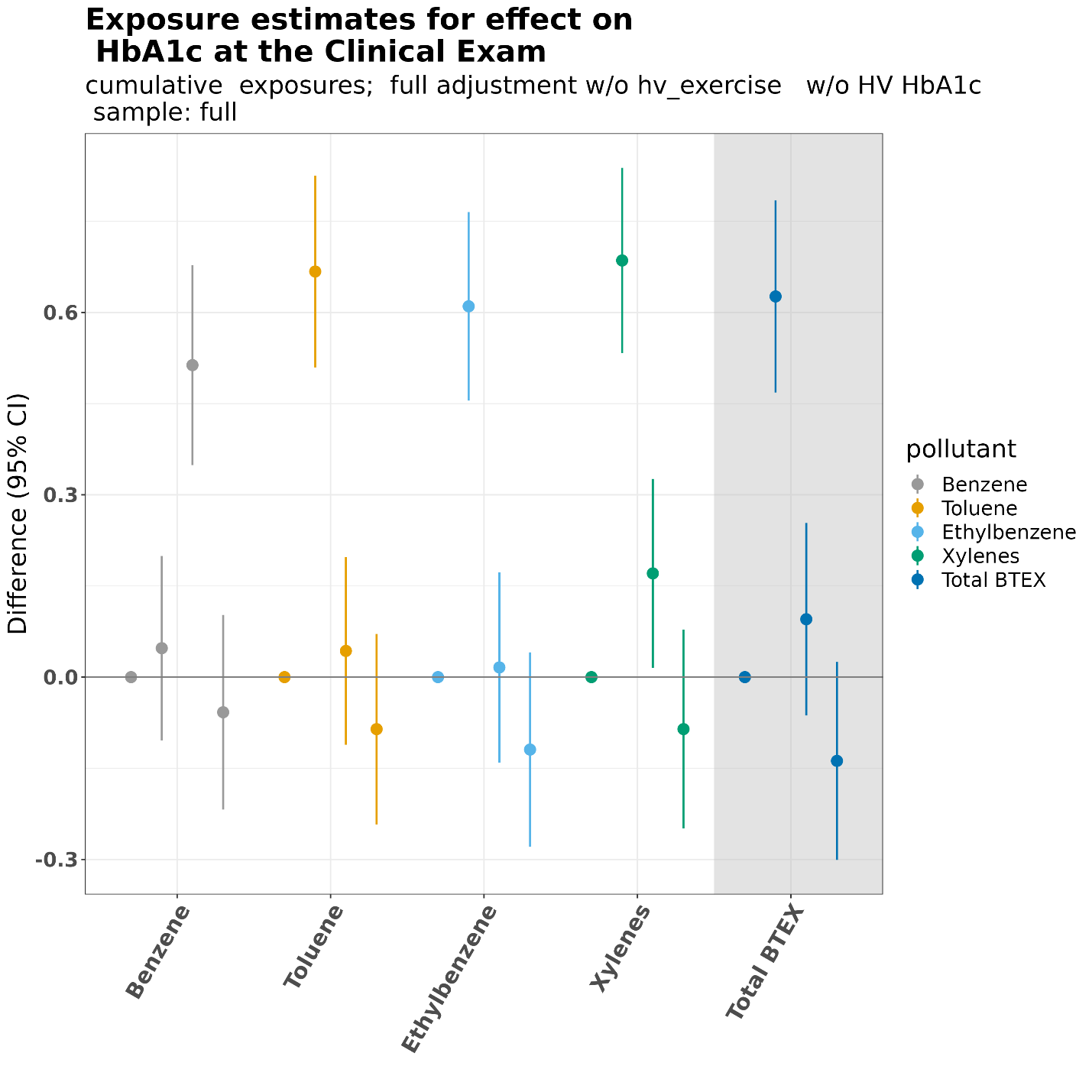


Supplementary Figure 6: Fully adjusted results (estimates and 95% confidence intervals) examining mean difference in Clinical Exam (final) HbA1c (%) by quartile of cumulative exposure when compared to Q1, not controlling for Home Visit (initial) HbA1c.

Supplementary Table 1: Variables used in calculation of inverse probability of censoring (treatment with HbA1c-lowering medication) weights

| Home Visit weights | Numerator | Age at enrollment + BMI at Home Visit |
| --- | --- | --- |
|  | Denominator | Age at enrollment + BMI at Home Visit + Health Insurance Status at Home Visit + Education at Enrollment + Race + Sex + Smoking status at Enrollment + Potential previous occupational exposure + Age at enrollment*Race + BMI at Home Visit*Race |
| Clinical Exam weights | Numerator | Age at enrollment + BMI at Home Visit + BMI at Clinical Exam |
|  | Denominator | Age at enrollment + HbA1c at Home Visit + BMI at Clinical Exam + BMI at Home Visit + Diabetes medication at Home Visit + Health Insurance Status at Home Visit + Education at Enrollment + Race + Sex + Smoking status at Enrollment + Potential previous occupational exposure + Age at enrollment*Race + BMI at Clinical Exam*Race + BMI at Home Visit*Race + HbA1c at Home Visit*Race |

Supplementary Table 2: Results from fully adjusted analyses examining Home Visit Exam HbA1c (%)

| **Exposure** | **Quartile** | **Difference (95% CI)** | **CLD** |
| --- | --- | --- | --- |
| Benzene | 1 | 1 (ref) | - |
|  | 2 | 0.07 (-0.05, 0.18) | 0.24 |
|  | 3 | 0.05 (-0.05, 0.16) | 0.21 |
|  | 4 | 0.03 (-0.08, 0.14) | 0.22 |
| Toluene | 1 | 1 (ref) | - |
|  | 2 | 0.07 (-0.05, 0.18) | 0.23 |
|  | 3 | 0.02 (-0.09, 0.13) | 0.22 |
|  | 4 | 0.04 (-0.07, 0.14) | 0.21 |
| Ethylbenzene | 1 | 1 (ref) | - |
|  | 2 | 0.03 (-0.08, 0.14) | 0.22 |
|  | 3 | 0.06 (-0.05, 0.16) | 0.22 |
|  | 4 | 0.03 (-0.08, 0.14) | 0.22 |
| Xylenes | 1 | 1 (ref) | - |
|  | 2 | 0.03 (-0.08, 0.14) | 0.22 |
|  | 3 | 0.11 (-0.01, 0.23) | 0.24 |
|  | 4 | 0.01 (-0.10, 0.12) | 0.21 |
| Total BTEX | 1 | 1 (ref) | - |
|  | 2 | 0.05 (-0.07, 0.16) | 0.23 |
|  | 3 | 0.07 (-0.04, 0.19) | 0.23 |
|  | 4 | 0.01 (-0.09, 0.12) | 0.21 |
| Mixture* | 1 | 0.13 (-0.16, 0.43) | 0.59 |
| *Mixture estimate is from Quantile G-Computation analysis and represents the estimate associated with a simultaneous 3-quartile increase in all constituent chemicals (BTEX) | | | |

Supplementary Table 3: Results from age adjusted analyses examining Clinical Exam HbA1c (%), controlling for Home Visit HbA1c

| **Exposure** | **Quartile** | **Difference (95% CI)** | **CLD** |
| --- | --- | --- | --- |
| Benzene | 1 | 1 (ref) | - |
|  | 2 | 0.03 (-0.07, 0.13) | 0.19 |
|  | 3 | 0.09 (-0.01, 0.19) | 0.21 |
|  | 4 | -0.01 (-0.10, 0.08) | 0.19 |
| Toluene | 1 | 1 (ref) | - |
|  | 2 | 0.25 (0.15, 0.34) | 0.20 |
|  | 3 | 0.11 (0.01, 0.21) | 0.20 |
|  | 4 | -0.03 (-0.12, 0.06) | 0.18 |
| Ethylbenzene | 1 | 1 (ref) | - |
|  | 2 | 0.23 (0.13, 0.33) | 0.20 |
|  | 3 | 0.11 (0.02, 0.21) | 0.19 |
|  | 4 | 0.04 (-0.06, 0.13) | 0.19 |
| Xylenes | 1 | 1 (ref) | - |
|  | 2 | 0.14 (0.05, 0.24) | 0.19 |
|  | 3 | 0.14 (0.04, 0.24) | 0.19 |
|  | 4 | -0.01 (-0.10, 0.09) | 0.19 |
| Total BTEX | 1 | 1 (ref) | - |
|  | 2 | 0.25 (0.15, 0.35) | 0.20 |
|  | 3 | 0.14 (0.04, 0.24) | 0.20 |
|  | 4 | -0.02 (-0.12, 0.07) | 0.19 |
| Mixture* | 1 | -0.06 (-0.14, 0.03) | 0.17 |
| *Mixture estimate is from Quantile G-Computation analysis and represents the estimate associated with a simultaneous 3-quartile increase in all constituent chemicals (BTEX) | | | |

Supplementary Table 4: Results from fully adjusted (main) analyses examining Clinical Exam HbA1c (%), controlling for Home Visit HbA1c

| **Exposure** | **Quartile** | **Difference (95% CI)** | **CLD** |
| --- | --- | --- | --- |
| Benzene | 1 | 1 (ref) | - |
|  | 2 | 0.06 (-0.03, 0.16) | 0.20 |
|  | 3 | 0.11 (-0.00, 0.21) | 0.21 |
|  | 4 | 0.01 (-0.09, 0.12) | 0.21 |
| Toluene | 1 | 1 (ref) | - |
|  | 2 | 0.24 (0.14, 0.34) | 0.20 |
|  | 3 | 0.12 (0.02, 0.22) | 0.20 |
|  | 4 | -0.01 (-0.11, 0.09) | 0.20 |
| Ethylbenzene | 1 | 1 (ref) | - |
|  | 2 | 0.22 (0.12, 0.32) | 0.20 |
|  | 3 | 0.11 (0.01, 0.21) | 0.20 |
|  | 4 | 0.05 (-0.05, 0.15) | 0.20 |
| Xylenes | 1 | 1 (ref) | - |
|  | 2 | 0.14 (0.05, 0.24) | 0.19 |
|  | 3 | 0.14 (0.04, 0.24) | 0.20 |
|  | 4 | 0.01 (-0.09, 0.12) | 0.21 |
| Total BTEX | 1 | 1 (ref) | - |
|  | 2 | 0.24 (0.14, 0.34) | 0.20 |
|  | 3 | 0.13 (0.03, 0.24) | 0.20 |
|  | 4 | -0.00 (-0.11, 0.10) | 0.21 |
| Mixture* | 1 | -0.04 (-0.13, 0.05) | 0.18 |
| *Mixture estimate is from Quantile G-Computation analysis and represents the estimate associated with a simultaneous 3-quartile increase in all constituent chemicals (BTEX) | | | |

Supplementary Table 5: Results from fully adjusted (main) analyses examining Clinical Exam HbA1c (%), controlling for Home Visit HbA1c, stratified by body mass index category

|  |  | BMI < 25 | |  | 25≤BMI<30 | |  | BMI ≥30 | |
| --- | --- | --- | --- | --- | --- | --- | --- | --- | --- |
| **Exposure** | **Quartile** | **Difference**  **(95% CI)** | **CLD** |  | **Difference**  **(95% CI)** | **CLD** |  | **Difference**  **(95% CI)** | **CLD** |
| Benzene | 1 | 1 (ref) | - |  | 1 (ref) | - |  | 1 (ref) | - |
|  | 2 | 0.02 (-0.06, 0.10) | 0.15 |  | 0.01 (-0.08, 0.11) | 0.19 |  | 0.15 (-0.06, 0.37) | 0.43 |
|  | 3 | -0.04 (-0.17, 0.09) | 0.26 |  | -0.06 (-0.21, 0.09) | 0.30 |  | 0.32 (0.09, 0.54) | 0.45 |
|  | 4 | -0.06 (-0.14, 0.02) | 0.16 |  | -0.01 (-0.12, 0.09) | 0.21 |  | 0.10 (-0.14, 0.33) | 0.47 |
| Toluene | 1 | 1 (ref) | - |  | 1 (ref) | - |  | 1 (ref) | - |
|  | 2 | 0.23 (0.14, 0.33) | 0.18 |  | 0.06 (-0.05, 0.18) | 0.23 |  | 0.39 (0.18, 0.60) | 0.42 |
|  | 3 | 0.07 (-0.06, 0.21) | 0.27 |  | -0.10 (-0.23, 0.02) | 0.25 |  | 0.31 (0.09, 0.54) | 0.45 |
|  | 4 | 0.05 (-0.03, 0.14) | 0.17 |  | -0.04 (-0.16, 0.07) | 0.22 |  | 0.03 (-0.20, 0.25) | 0.45 |
| Ethylbenzene | 1 | 1 (ref) | - |  | 1 (ref) | - |  | 1 (ref) | - |
|  | 2 | 0.20 (0.11, 0.29) | 0.18 |  | 0.03 (-0.08, 0.14) | 0.23 |  | 0.34 (0.13, 0.55) | 0.42 |
|  | 3 | 0.06 (-0.09, 0.20) | 0.29 |  | -0.01 (-0.13, 0.10) | 0.23 |  | 0.23 (0.02, 0.45) | 0.43 |
|  | 4 | 0.07 (-0.01, 0.15) | 0.16 |  | -0.07 (-0.18, 0.05) | 0.24 |  | 0.12 (-0.10, 0.35) | 0.45 |
| Xylenes | 1 | 1 (ref) | - |  | 1 (ref) | - |  | 1 (ref) | - |
|  | 2 | -0.02 (-0.10, 0.07) | 0.17 |  | 0.03 (-0.08, 0.14) | 0.22 |  | 0.33 (0.13, 0.53) | 0.40 |
|  | 3 | -0.03 (-0.15, 0.08) | 0.23 |  | -0.01 (-0.12, 0.11) | 0.23 |  | 0.31 (0.10, 0.52) | 0.42 |
|  | 4 | -0.03 (-0.11, 0.05) | 0.16 |  | -0.06 (-0.18, 0.07) | 0.25 |  | 0.12 (-0.10, 0.33) | 0.43 |
| Total BTEX | 1 | 1 (ref) | - |  | 1 (ref) | - |  | 1 (ref) | - |
|  | 2 | 0.20 (0.11, 0.29) | 0.18 |  | 0.08 (-0.04, 0.19) | 0.23 |  | 0.33 (0.12, 0.54) | 0.42 |
|  | 3 | 0.07 (-0.06, 0.20) | 0.27 |  | -0.07 (-0.19, 0.05) | 0.23 |  | 0.30 (0.08, 0.52) | 0.45 |
|  | 4 | 0.06 (-0.02, 0.14) | 0.16 |  | -0.09 (-0.21, 0.04) | 0.25 |  | 0.04 (-0.19, 0.27) | 0.46 |
| Mixture* | 1 | -0.02 (-0.09, 0.06) | 0.15 |  | -0.04 (-0.14, 0.07) | 0.21 |  | -0.07 (-0.26, 0.12) | 0.38 |
| *Mixture estimate is from Quantile G-Computation analysis and represents the estimate associated with a simultaneous 3-quartile increase in all constituent chemicals (BTEX) | | | | | | | | | |

Supplementary Table 6: Results from fully adjusted (main) analyses examining Clinical Exam HbA1c (%), controlling for Home Visit HbA1c, stratified by Area Deprivation Index

|  |  | **ADI Q1/2** | |  | **ADI Q3** | |  | **ADI Q4** | |
| --- | --- | --- | --- | --- | --- | --- | --- | --- | --- |
| **Exposure** | **Quartile** | **Difference  (95% CI)** | **CLD** |  | **Difference  (95% CI)** | **CLD** |  | **Difference  (95% CI)** | **CLD** |
| Benzene | 1 | 1 (ref) | - |  | 1 (ref) | - |  | 1 (ref) | - |
|  | 2 | -0.01 (-0.19, 0.17) | 0.36 |  | 0.28 (0.06, 0.49) | 0.44 |  | -0.03 (-0.17, 0.10) | 0.27 |
|  | 3 | 0.17 (-0.02, 0.37) | 0.39 |  | 0.00 (-0.23, 0.24) | 0.48 |  | 0.10 (-0.03, 0.24) | 0.27 |
|  | 4 | 0.14 (-0.06, 0.35) | 0.40 |  | -0.05 (-0.29, 0.19) | 0.47 |  | 0.01 (-0.13, 0.15) | 0.29 |
| Toluene | 1 | 1 (ref) | - |  | 1 (ref) | - |  | 1 (ref) | - |
|  | 2 | 0.03 (-0.13, 0.20) | 0.34 |  | 0.37 (0.14, 0.59) | 0.45 |  | 0.23 (0.10, 0.37) | 0.27 |
|  | 3 | 0.22 (0.02, 0.42) | 0.40 |  | -0.10 (-0.32, 0.13) | 0.44 |  | 0.09 (-0.04, 0.22) | 0.26 |
|  | 4 | -0.00 (-0.19, 0.18) | 0.37 |  | -0.10 (-0.34, 0.14) | 0.48 |  | 0.10 (-0.04, 0.24) | 0.28 |
| Ethylbenzene | 1 | 1 (ref) | - |  | 1 (ref) | - |  | 1 (ref) | - |
|  | 2 | 0.01 (-0.15, 0.17) | 0.32 |  | 0.29 (0.06, 0.51) | 0.45 |  | 0.23 (0.10, 0.37) | 0.27 |
|  | 3 | 0.22 (0.06, 0.38) | 0.31 |  | 0.02 (-0.21, 0.25) | 0.46 |  | 0.07 (-0.07, 0.20) | 0.26 |
|  | 4 | 0.08 (-0.08, 0.24) | 0.32 |  | -0.06 (-0.31, 0.18) | 0.49 |  | 0.08 (-0.05, 0.20) | 0.26 |
| Xylenes | 1 | 1 (ref) | - |  | 1 (ref) | - |  | 1 (ref) | - |
|  | 2 | -0.01 (-0.18, 0.16) | 0.34 |  | 0.30 (0.10, 0.50) | 0.40 |  | 0.11 (-0.04, 0.25) | 0.28 |
|  | 3 | 0.22 (0.03, 0.40) | 0.37 |  | 0.04 (-0.17, 0.24) | 0.41 |  | 0.07 (-0.08, 0.21) | 0.29 |
|  | 4 | 0.01 (-0.18, 0.21) | 0.39 |  | 0.02 (-0.20, 0.24) | 0.45 |  | 0.03 (-0.11, 0.16) | 0.27 |
| Total BTEX | 1 | 1 (ref) | - |  | 1 (ref) | - |  | 1 (ref) | - |
|  | 2 | 0.03 (-0.14, 0.19) | 0.32 |  | 0.34 (0.12, 0.57) | 0.45 |  | 0.24 (0.10, 0.38) | 0.27 |
|  | 3 | 0.23 (0.05, 0.41) | 0.36 |  | -0.07 (-0.29, 0.16) | 0.45 |  | 0.13 (-0.00, 0.27) | 0.27 |
|  | 4 | 0.04 (-0.14, 0.23) | 0.37 |  | -0.08 (-0.33, 0.16) | 0.49 |  | 0.05 (-0.07, 0.18) | 0.25 |
| Mixture* | 1 | 0.00 (-0.16, 0.16) | 0.32 |  | -0.18 (-0.37, 0.00) | 0.37 |  | 0.04 (-0.08, 0.16) | 0.23 |
| *Mixture estimate is from Quantile G-Computation analysis and represents the estimate associated with a simultaneous 3-quartile increase in all constituent chemicals (BTEX) | | | | | | | | | |

Supplementary Table 7: Results from fully adjusted (main) analyses examining Clinical Exam HbA1c (%), controlling for Home Visit HbA1c, stratified by self-classified race

|  |  | white | |  | Black | |
| --- | --- | --- | --- | --- | --- | --- |
| **Exposure** | **Quartile** | **Difference  (95% CI)** | **CLD** |  | **Difference  (95% CI)** | **CLD** |
| Benzene | 1 | 1 (ref) | - |  | 1 (ref) | - |
|  | 2 | 0.09 (-0.05, 0.23) | 0.28 |  | -0.02 (-0.17, 0.12) | 0.29 |
|  | 3 | -0.04 (-0.18, 0.10) | 0.28 |  | 0.26 (0.08, 0.44) | 0.36 |
|  | 4 | 0.06 (-0.09, 0.21) | 0.31 |  | -0.08 (-0.23, 0.07) | 0.31 |
| Toluene | 1 | 1 (ref) | - |  | 1 (ref) | - |
|  | 2 | 0.31 (0.17, 0.44) | 0.27 |  | 0.14 (-0.01, 0.29) | 0.30 |
|  | 3 | 0.11 (-0.02, 0.24) | 0.26 |  | 0.10 (-0.08, 0.27) | 0.34 |
|  | 4 | 0.01 (-0.14, 0.15) | 0.28 |  | -0.04 (-0.19, 0.11) | 0.30 |
| Ethylbenzene | 1 | 1 (ref) | - |  | 1 (ref) | - |
|  | 2 | 0.25 (0.12, 0.38) | 0.27 |  | 0.13 (-0.03, 0.28) | 0.31 |
|  | 3 | 0.05 (-0.07, 0.18) | 0.25 |  | 0.18 (0.02, 0.34) | 0.32 |
|  | 4 | 0.09 (-0.04, 0.22) | 0.26 |  | -0.08 (-0.24, 0.07) | 0.31 |
| Xylenes | 1 | 1 (ref) | - |  | 1 (ref) | - |
|  | 2 | 0.10 (-0.03, 0.23) | 0.26 |  | 0.10 (-0.05, 0.26) | 0.31 |
|  | 3 | 0.08 (-0.05, 0.20) | 0.25 |  | 0.18 (0.02, 0.34) | 0.32 |
|  | 4 | 0.03 (-0.11, 0.17) | 0.28 |  | -0.06 (-0.24, 0.12) | 0.36 |
| Total BTEX | 1 | 1 (ref) | - |  | 1 (ref) | - |
|  | 2 | 0.30 (0.17, 0.43) | 0.27 |  | 0.12 (-0.03, 0.27) | 0.30 |
|  | 3 | 0.11 (-0.02, 0.25) | 0.27 |  | 0.14 (-0.04, 0.31) | 0.35 |
|  | 4 | 0.06 (-0.08, 0.20) | 0.28 |  | -0.09 (-0.23, 0.06) | 0.28 |
| Mixture* | 1 | -0.04 (-0.15, 0.08) | 0.23 |  | -0.05 (-0.19, 0.09) | 0.28 |
| *Mixture estimate is from Quantile G-Computation analysis and represents the estimate associated with a simultaneous 3-quartile increase in all constituent chemicals (BTEX) | | | | | | |

Supplementary Table 8: Descriptive statistics of the analysis sample by cumulative total BTEX quartile

|  |  | Cumulative total BTEX quartile | | | |
| --- | --- | --- | --- | --- | --- |
| **Characteristic** | **Overall**, N = 2,541*^1^* | **1**, N = 636*^1^* | **2**, N = 635*^1^* | **3**, N = 635*^1^* | **4**, N = 635*^1^* |
| **Age (months)** | 558 (423, 660) | 572 (431, 671) | 568 (431, 668) | 565 (419, 653) | 544 (413, 637) |
| missing | 0 | 0 | 0 | 0 | 0 |
| **Race** |  |  |  |  |  |
| Black | 1,015 (40%) | 272 (43%) | 285 (45%) | 230 (36%) | 228 (36%) |
| Other | 199 (7.8%) | 51 (8.0%) | 37 (5.8%) | 52 (8.2%) | 59 (9.3%) |
| white | 1,327 (52%) | 313 (49%) | 313 (49%) | 353 (56%) | 348 (55%) |
| missing | 0 | 0 | 0 | 0 | 0 |
| **Sex** |  |  |  |  |  |
| male | 1,979 (78%) | 404 (64%) | 469 (74%) | 535 (84%) | 571 (90%) |
| female | 562 (22%) | 232 (36%) | 166 (26%) | 100 (16%) | 64 (10%) |
| missing | 0 | 0 | 0 | 0 | 0 |
| **Educational attainment** |  |  |  |  |  |
| less than HS | 554 (22%) | 117 (18%) | 121 (19%) | 154 (24%) | 162 (26%) |
| HS/GED | 862 (34%) | 198 (31%) | 217 (34%) | 231 (36%) | 216 (34%) |
| some college | 781 (31%) | 208 (33%) | 203 (32%) | 188 (30%) | 182 (29%) |
| 4 yr college/more | 344 (14%) | 113 (18%) | 94 (15%) | 62 (9.8%) | 75 (12%) |
| missing | 0 | 0 | 0 | 0 | 0 |
| **Employment** |  |  |  |  |  |
| other | 372 (15%) | 115 (18%) | 95 (15%) | 89 (14%) | 73 (12%) |
| unemployed | 749 (30%) | 168 (26%) | 208 (33%) | 185 (29%) | 188 (30%) |
| working/student/keeping house | 1,414 (56%) | 353 (56%) | 331 (52%) | 358 (57%) | 372 (59%) |
| missing | 6 | 0 | 1 | 3 | 2 |
| **Smoking status** |  |  |  |  |  |
| former smoker | 562 (22%) | 145 (23%) | 138 (22%) | 142 (23%) | 137 (22%) |
| heavy current smoker | 277 (11%) | 53 (8.4%) | 71 (11%) | 79 (13%) | 74 (12%) |
| light current smoker | 570 (23%) | 122 (19%) | 148 (24%) | 136 (22%) | 164 (26%) |
| never smoked | 1,098 (44%) | 309 (49%) | 269 (43%) | 272 (43%) | 248 (40%) |
| missing | 34 | 7 | 9 | 6 | 12 |
| **Potential prior exposure to BTEX** |  |  |  |  |  |
| 0 | 963 (38%) | 293 (46%) | 264 (42%) | 214 (34%) | 192 (30%) |
| 1 | 1,576 (62%) | 342 (54%) | 371 (58%) | 421 (66%) | 442 (70%) |
| missing | 2 | 1 | 0 | 0 | 1 |
| **Body Mass Index (kg/m^2)** | 29 (25, 34) | 29 (25, 34) | 29 (25, 34) | 29 (26, 33) | 29 (25, 33) |
| missing | 19 | 6 | 6 | 2 | 5 |
| **ADI quartile** |  |  |  |  |  |
| 1/2 | 721 (28%) | 183 (29%) | 173 (27%) | 185 (29%) | 180 (28%) |
| 3 | 888 (35%) | 197 (31%) | 221 (35%) | 245 (39%) | 225 (35%) |
| 4 | 921 (36%) | 251 (40%) | 239 (38%) | 202 (32%) | 229 (36%) |
| missing | 11 | 5 | 2 | 3 | 1 |
| **Health insurance status** |  |  |  |  |  |
| no | 1,260 (50%) | 269 (43%) | 307 (49%) | 332 (53%) | 352 (56%) |
| yes | 1,246 (50%) | 361 (57%) | 321 (51%) | 291 (47%) | 273 (44%) |
| missing | 35 | 6 | 7 | 12 | 10 |
| **Cumulative benzene (ppb)** | 499 (151, 1,258) | 44 (11, 99) | 314 (208, 436) | 825 (569, 1,168) | 1,987 (1,351, 3,049) |
| missing | 0 | 0 | 0 | 0 | 0 |
| **Cumulative toluene(ppb)** | 2,107 (575, 4,829) | 154 (60, 361) | 1,226 (853, 1,712) | 3,375 (2,668, 4,118) | 7,502 (5,623, 10,109) |
| missing | 0 | 0 | 0 | 0 | 0 |
| **Cumulative ethylbenzene(ppb)** | 365 (123, 877) | 36 (8, 76) | 236 (170, 315) | 587 (424, 803) | 1,476 (994, 2,066) |
| missing | 0 | 0 | 0 | 0 | 0 |
| **Cumulative xylenes(ppb)** | 2,466 (1,065, 4,970) | 519 (239, 822) | 1,706 (1,371, 2,123) | 3,622 (2,848, 4,418) | 7,202 (5,698, 9,363) |
| missing | 0 | 0 | 0 | 0 | 0 |
| **Cumulative total BTEX(ppb)** | 5,362 (1,919, 11,300) | 832 (377, 1,314) | 3,429 (2,704, 4,348) | 8,005 (6,503, 9,616) | 16,343 (13,299, 21,551) |
| missing | 0 | 0 | 0 | 0 | 0 |
| *^1^*Median (IQR); n (%) | | | | | |

Supplementary Table 9: Descriptions of potential selection factors for all GuLF Study workers by quartile of cumulative total BTEX

|  |  | **Quartile of cumulative BTEX exposure** | | | | |
| --- | --- | --- | --- | --- | --- | --- |
| **Characteristic** | **Overall**, N = 24,375*^1^* | **1**, N = 8,576*^1^* | **3**, N = 4,856*^1^* | **2**, N = 5,668*^1^* | **4**, N = 5,076*^1^* | **missing**, N = 199*^1^* |
| Completed Home Visit |  |  |  |  |  |  |
| no | 15,407 (63%) | 6,057 (71%) | 2,948 (61%) | 3,452 (61%) | 2,807 (55%) | 143 (72%) |
| yes | 8,968 (37%) | 2,519 (29%) | 1,908 (39%) | 2,216 (39%) | 2,269 (45%) | 56 (28%) |
| missing | 0 | 0 | 0 | 0 | 0 | 0 |
| Completed Clinical Exam |  |  |  |  |  |  |
| no | 1,766 (38%) | 447 (39%) | 395 (37%) | 434 (38%) | 476 (37%) | 14 (40%) |
| yes | 2,873 (62%) | 685 (61%) | 667 (63%) | 696 (62%) | 804 (63%) | 21 (60%) |
| missing | 19,736 | 7,444 | 3,794 | 4,538 | 3,796 | 164 |
| eligible for CE |  |  |  |  |  |  |
| no | 19,736 (81%) | 7,444 (87%) | 3,794 (78%) | 4,538 (80%) | 3,796 (75%) | 164 (82%) |
| yes | 4,639 (19%) | 1,132 (13%) | 1,062 (22%) | 1,130 (20%) | 1,280 (25%) | 35 (18%) |
| missing | 0 | 0 | 0 | 0 | 0 | 0 |
| eligible for CE and CE done |  |  |  |  |  |  |
| CE done | 2,873 (12%) | 685 (8.0%) | 667 (14%) | 696 (12%) | 804 (16%) | 21 (11%) |
| CE not done | 1,766 (7.2%) | 447 (5.2%) | 395 (8.1%) | 434 (7.7%) | 476 (9.4%) | 14 (7.0%) |
| not eligible | 19,736 (81%) | 7,444 (87%) | 3,794 (78%) | 4,538 (80%) | 3,796 (75%) | 164 (82%) |
| missing | 0 | 0 | 0 | 0 | 0 | 0 |
| Death (any cause) |  |  |  |  |  |  |
| 0 | 22,700 (93%) | 8,066 (94%) | 4,503 (93%) | 5,272 (93%) | 4,677 (92%) | 182 (91%) |
| 1 | 1,675 (6.9%) | 510 (5.9%) | 353 (7.3%) | 396 (7.0%) | 399 (7.9%) | 17 (8.5%) |
| missing | 0 | 0 | 0 | 0 | 0 | 0 |
| Death (diabetes listed) |  |  |  |  |  |  |
| 0 | 24,232 (99%) | 8,531 (99%) | 4,819 (99%) | 5,638 (99%) | 5,047 (99%) | 197 (99%) |
| 1 | 143 (0.6%) | 45 (0.5%) | 37 (0.8%) | 30 (0.5%) | 29 (0.6%) | 2 (1.0%) |
| missing | 0 | 0 | 0 | 0 | 0 | 0 |
| *^1^*n (%) | | | | | | |
